# Vincristine-Induced Peripheral Neuropathy in Pediatric Oncology: A Randomized Controlled Trial Comparing Push Injections with One-Hour Infusions (The VINCA Trial)

**DOI:** 10.3390/cancers12123745

**Published:** 2020-12-12

**Authors:** Mirjam Esther van de Velde, Gertjan J. L. Kaspers, Floor C. H. Abbink, Jos W. R. Twisk, Inge M. van der Sluis, Cor van den Bos, Marry M. van den Heuvel-Eibrink, Heidi Segers, Christophe Chantrain, Jutte van der Werff ten Bosch, Leen Willems, Marleen H. van den Berg

**Affiliations:** 1 Emma Children’s Hospital, Amsterdam UMC, Vrije Universiteit Amsterdam, Pediatric Oncology, 1081 HV Amsterdam, The Netherlands; g.j.l.kaspers@prinsesmaximacentrum.nl (G.J.L.K.); mh.vandenberg@amsterdamumc.nl (M.H.v.d.B.); 2 Princess Máxima Center for Pediatric Oncology, 3584 CS Utrecht, The Netherlands; I.M.vanderSluis@prinsesmaximacentrum.nl (I.M.v.d.S.); C.vandenBos-5@prinsesmaximacentrum.nl (C.v.d.B.); m.m.vandenheuvel-eibrink@prinsesmaximacentrum.nl (M.M.v.d.H.-E.); 3 Emma Children’s Hospital, Amsterdam UMC, Amsterdam Medical Center, Pediatric Oncology, 1105 AZ Amsterdam, The Netherlands; f.abbink@amsterdamumc.nl; 4 Department of Epidemiology and Biostatistics, Amsterdam UMC, Vrije Universiteit Amsterdam, 1081 HV Amsterdam, The Netherlands; jwr.twisk@amsterdamumc.nl; 5 Department of Pediatric Oncology, Erasmus Medical Center Rotterdam/Sophia Children’s Hospital, 3000 CB Rotterdam, The Netherlands; 6 Department of Pediatric Hemato-Oncology, University Hospitals Leuven, 3000 Leuven, Belgium; heidi.segers@uzleuven.be; 7 Department of Pediatrics, Clinique du MontLégia, CHC, 4000 Liège, Belgium; christophe.chantrain@chc.be; 8 Department of Pediatric Onco-Hematology, Universitair Ziekenhuis Brussel, 1090 Brussels, Belgium; jutte.VanderWerffTenBosch@uzbrussel.be; 9 Department of Paediatric Haematology-Oncology and Stem Cell Transplantation, Ghent University Hospital, 9000 Ghent, Belgium; leen.willems@uzgent.be

**Keywords:** neurotoxicity, exposure, children, cancer, vincristine, toxicity, administration duration, infusion rate, adolescent, chemotherapeutic, oncovin

## Abstract

**Simple Summary:**

We studied children with cancer who receive vincristine chemotherapy as a one-hour infusion and compared them to children who received vincristine as a push injection. We investigated if there was a difference in the development of vincristine induced peripheral neuropathy, the most common side effect of vincristine. We found that in general there were no differences between the two groups (one-hour group versus push group). However, in children using both vincristine and azole antifungals, we noticed that children who received vincristine as a one-hour infusion experienced less severe vincristine induced peripheral neuropathy compared to children who received vincristine as a push injection. Therefore, in children who require both azole antifungal medication as well as vincristine it might be beneficial to administer the vincristine as a one-hour infusion instead of a push injection. However, this finding needs to be confirmed in other studies as well.

**Abstract:**

Vincristine (VCR) is a frequently used chemotherapeutic agent. However, it can lead to VCR-induced peripheral neuropathy (VIPN). In this study we investigated if one-hour infusions of VCR instead of push-injections reduces VIPN in pediatric oncology patients. We conducted a multicenter randomized controlled trial in which participants received all VCR administrations through push injections or one-hour infusions. VIPN was measured at baseline and 1–5 times during treatment using Common Terminology Criteria of Adverse Events (CTCAE) and pediatric-modified Total Neuropathy Score. Moreover, data on co-medication, such as azole antifungals, were collected. Overall, results showed no effect of administration duration on total CTCAE score or ped-mTNS score. However, total CTCAE score was significantly lower in patients receiving one-hour infusions concurrently treated with azole antifungal therapy (β = -1.58; *p* = 0.04). In conclusion, generally VCR administration through one-hour infusions does not lead to less VIPN compared to VCR push injections in pediatric oncology patients. However, one-hour infusions lead to less severe VIPN compared to push-injections when azole therapy is administered concurrently with VCR. These results indicate that in children treated with VCR and requiring concurrent azole therapy, one-hour infusions might be beneficial over push injections, although larger trials are needed to confirm this association.

## 1. Introduction

Vincristine (VCR) is a frequently-used chemotherapeutic agent in pediatric oncology since many decades [1,2]. VCR inhibits the mitotic spindle in the cell, thereby blocking cell division [3,4]. In the liver, it is metabolized into M1 by the cytochrome P450 (CYP) 3A enzymes [5]. A major adverse effect of VCR is neurotoxicity, which is characterized by autonomic and peripheral sensory-motor neuropathy and reported in 12–87% of VCR-exposed children [1,6,7,8]. Symptoms of VCR-induced peripheral neuropathy (VIPN) include paresthesia, muscle weakness, areflexia, pain, and diminished sensibility [9,10,11,12]. It usually starts after a few administrations and symptoms often reside several months after treatment cessation [1,2]. VIPN can lead to suboptimal treatment due to dose reductions or omissions of VCR [10,13]. VIPN is dose-dependent, with single administration doses exceeding 2.0 mg/m^2^ leading to intolerable VIPN in children [14]. Children of older age or Caucasian ancestry seem more vulnerable. Furthermore, genetic predispositions and pharmacokinetics (PK) of VCR influence VIPN development [1,6,14,15,16,17,18,19,20,21]. Moreover, multiple studies have shown that concurrent azole antifungal and VCR treatment leads to more and severe VIPN in children, due to competitive interaction by the CYP enzyme [22,23,24]. This is clinically relevant, since azole antifungals are frequently used to prevent or treat invasive fungal infections in pediatric cancer [25]. Although VIPN is dose-limiting [1,14], prolongation of VCR administration showed that the single administration dose can be increased without leading to intolerable VIPN in children. In two studies using continuous VCR infusion up to five days, cumulative VCR doses of 4.0 mg/m^2^ were well tolerated [26,27]. This could be due to lower peak-plasma concentrations that are related to longer lasting infusions, which seem associated with less VIPN [28]. Yet, multiple day VCR infusions are costly and cumbersome. Therefore, in clinical practice VCR is usually administered intravenously (iv) through a short-term push injection or infusion (up to 15 min). Sometimes it is administered through a one-hour infusion. Both push and one-hour administrations use standardized VCR doses of 1.5–2.0 mg/m^2^ (maximum 2.0 mg) [29]. However, the effect of prolonging VCR infusion on VIPN using standardized dosing regimens, is unknown. Therefore, we conducted a randomized controlled trial (RCT) to determine whether intravenous one-hour infusions of VCR are associated with less VIPN compared to intravenous push injections in pediatric oncology patients. In addition, we evaluated the potentially modifying effect of co-medication (i.e., concurrent azole antifungal treatment) on this association.

## 2. Results

### 2.1. Participants

**Figure 1:**
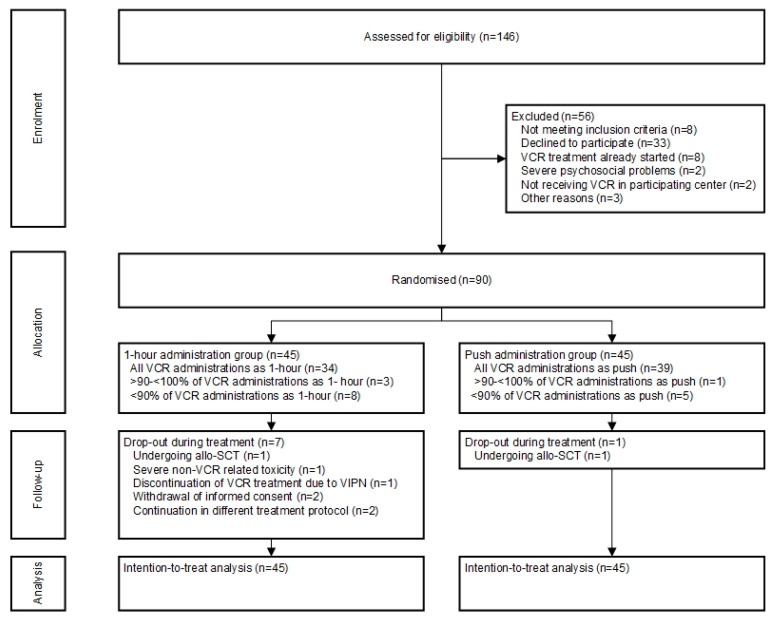
Flow diagram of screening, randomization, and follow-up. VCR: vincristine; allo-SCT: allogeneic stem-cell transplantation.

From September 2014 through January 2018, a total of 90 participants (*n* = 45 push administration, *n* = 45 one-hour administration) were enrolled (Figure 1). In general, baseline characteristics of the two groups were well-balanced (Table 1). Most participants were treated for acute lymphoblastic leukemia (ALL). Data of all 90 randomized patients were used for analysis. There was no difference in relapse rate (*p* > 0.99) or mortality (*p* = 0.62) between the two groups. In total, eight patients (9%) dropped-out during the trial, which was less than anticipated. Results of baseline peripheral neuropathy scores are reported in Table S1.

### 2.2. Primary Endpoints

**Figure 2:**
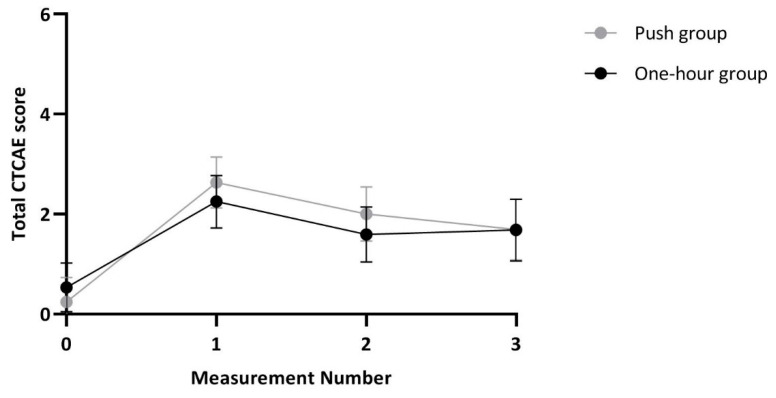
Total Common Terminology Criteria of Adverse Events score per administration method. CTCAE: Common Terminology Criteria of Adverse Events, dots represent the estimated values and lines of the 95% confidence interval of this estimate. Numbers per measurement: *t* = 0: *n* = 90, *t* = 1: *n* = 80, *t* = 2: *n* = 72, *t* = 3: 58. Although for *n* = 5 patients five measurements were available, results of the latter two of these measurements were not taken into account for this graph due to the small number of patients.

Overall, 43 out of 90 children developed VIPN as measured by Common Terminology Criteria of Adverse Events (CTCAE), of whom 9 had severe VIPN. Furthermore, according to three out of four CTCAE items more children in the push group were identified with VIPN compared to the one-hour group, although these differences were not statistically significant. Finally, 55 out of in total *n* = 66 patients aged ≥5 years developed VIPN based on pediatric modified Total Neuropathy Score (ped-mTNS) assessment of children ≥5 years of age. Results are summarized in Table 2. No statistically significant difference in VIPN as measured by the total CTCAE score, ped-mTNS score or use of analgesics between patients in both randomization groups was found (Figure 2 and Table 3).

**Figure 3:**
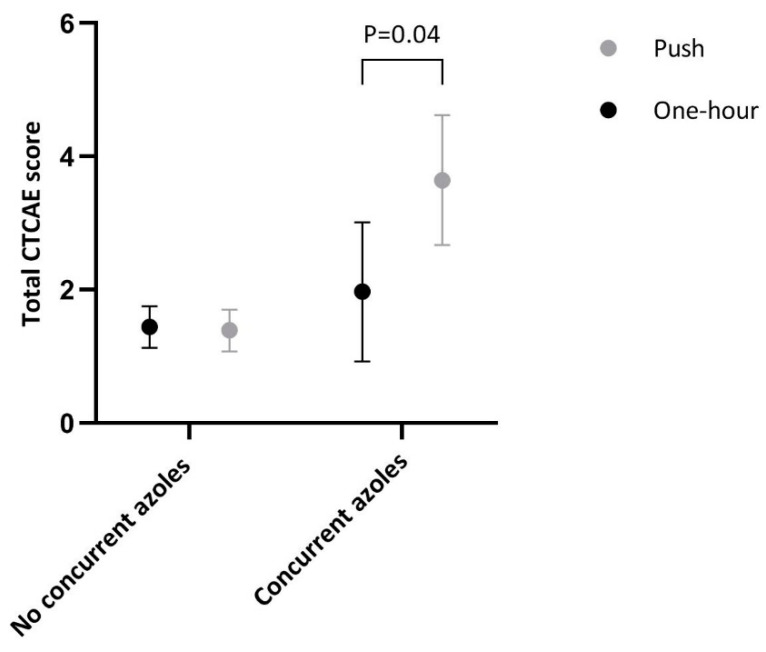
Total Common Terminology Criteria of Adverse Events score per randomization method and taking concurrent azole antifungal treatment into account. CTCAE: Common Terminology Criteria of Adverse Events, dots represent the estimated values and lines of the 95% confidence interval of this estimate. Numbers per group: no concurrent azoles: push group: *n* = 36, one-hour group: *n* = 40; concurrent azoles: push group: *n* = 9, one-hour group: *n* = 10.

Since concurrent azole treatment appeared to be an effect modifier (interaction term: *p* = 0.03), results are reported separately for measurements with (*n* = 19 patients) and without (*n* = 71 patients) concurrent azole treatment (Table 3 and Figure 3). Within the group of measurements with concurrent azole antifungal treatment (one-hour group: *n* = 10, push group: *n* = 9), total CTCAE scores were significantly lower in the one-hour group compared to the push group. Our results did not meaningfully change after adjustment for several covariates or when dichotomized outcomes were used (Table S2).

### 2.3. Secondary Endpoints

Analyses regarding the ped-mTNS provided similar results as those of CTCAE. Overall, no statistically significant effect of administration method on total ped-mTNS score was found. Again, when results were separately analyzed for measurements with concurrent azole treatment, there was a trend towards higher ped-mTNS scores in the push-administration group (Table 3). Results were comparable when additionally corrected or when using dichotomized ped-mTNS scores (Table S2). The risk of having VIPN measured by the use of analgesics was not significantly different for participants in the two randomization groups as well as within the two azole subgroups (Table 3 and Table S2).

## 3. Discussion

In the current study, in which 90 pediatric oncology patients were randomized to receive VCR administrations through iv push injections or one-hour infusions, overall VIPN did not differ between the two groups. However, when VCR was administered concurrently with azole antifungals, children in the one-hour group had a significantly lower total CTCAE score than those in the push group. When VIPN was assessed by ped-mTNS scores or by dichotomized outcomes (having VIPN or not), no significant differences were found between push administrations and one-hour infusions, irrespective of concurrent azole treatment, although a trend in the same direction as CTCAE results was shown.

VIPN is a debilitating toxicity with symptoms still present in adult survivors of childhood cancer [30]. Therefore, the overarching goal of this trial was to study an intervention possibly resulting in reduced VIPN during treatment of childhood cancer. To the best of our knowledge, this is the first RCT studying the effect of administration duration of VCR on VIPN, either in children or adults. During this trial we evaluated VIPN prospectively and longitudinally with repeated measurements. At each hospital uniformly trained assessors evaluated VIPN using two different instruments (CTCAE and ped-mTNS) including standardized physical examination [31]. Especially the ped-mTNS has been systematically reviewed and is currently recommended for the assessment of VIPN in children [32].

Our results show that one-hour infusions result in lower total CTCAE scores when VCR is concurrently used with azole antifungals. In general, this concurrent treatment is associated with increased incidence and severity of VIPN [22,23,24]. In our study, estimated CTCAE score was 1.41 (95%CI: 1.19 to 1.64) for measurements without concurrent azole treatment and more than twice as high (2.87 (95%CI: 2.15 to 3.58)) for measurements with concurrent azole treatment (*p* < 0.001). However, when these scores of VIPN with concurrent azole antifungal use were evaluated for patients in each randomization group separately, estimated CTCAE score of patients in the one-hour group was 1.97 (95%CI: 0.92 to 3.01) vs. 3.64 (95%CI: 2.67 to 4.62) in the push group for measurements with concurrent azole treatment. These results show that concurrent treatment of azole antifungals and VCR has a smaller impact on VIPN when VCR is administered through one-hour infusions. This could also well be true for concurrent use with other strong CYP3A inhibitors, such as anti-retroviral drugs or carbamazepine.

Since concurrent use of azole antifungals and VCR are generally avoided, alternative treatment for invasive fungal infections (IFI) must be used, such as echinocandins or (liposomal) amphotericin B. However, these agents have several disadvantages including high costs, iv administration only, and lack of evidence of superior efficacy over azole antifungals in children [33,34,35]. Therefore, in clinical practice IFI are frequently treated with azole antifungals irrespective of concurrent VCR treatment [36,37]. Although in clinical practice treatment with azole antifungals is sometimes discontinued 24 hours before VCR administration, this interval is too short, based on the half-life of azole antifungals, to have an impact on this drug-drug interaction [38]. It should be noted, that the absolute number of patients with concurrent VCR and azole treatment was small in our study (*n* = 19, 21%). Therefore, future studies should be undertaken to replicate our results and indisputably confirm the favorable effect of one-hour infusions over push injections regarding VIPN development. Furthermore, due to low patient numbers, we were not able to study the effect of azole type, such as itraconazole, fluconazole, voriconazole, and posaconazole. This could very well be of importance as itraconazole is a stronger inhibitor of CYP3A4 than for instance fluconazole or voriconazole [39]. Finally, diagnostic indication for treatment with azole antifungals, such as prophylaxis or treatment of diagnosed mycoses, on the relation between administration method and VIPN should be considered as well in a future study.

Although VIPN is a serious toxicity, high therapeutic effectiveness of VCR is of utmost importance. Regarding administration method, it is not expected that one-hour infusions of VCR are associated with a worse therapeutic outcome than push injections. It might even be the contrary. In general, longer lasting infusions of chemotherapy may improve therapeutic efficacy of a drug with a short half-life and an action mechanism of the drug that is related to the cell cycle, both of which are true for VCR [26]. While it is conceivable, that one-hour infusions are too short to benefit from this cell-cycle dependence or prolonged half-life, it underlines the unlikeliness of lower therapeutic effectiveness of one-hour infusions of VCR. In our study we also did not find any differences regarding relapse or mortality between the two groups.

Our study had some limitations. First of all, we included a heterogeneous group of patients with multiple diseases, varying VCR dosing regimens and co-medications. These different co-medications could theoretically alter pharmacokinetics and thus VCR exposure and VIPN development. In order to further investigate the true effect of VCR administration duration on the development of VIPN, future studies including more uniform diagnostic study groups are needed. Furthermore, in treatment protocols for ALL and Hodgkin’s lymphoma, the use of glucocorticoids is common practice [29,40]. Adverse effects of these agents could mimic symptoms of VIPN not attributed to VCR. However, we see a similar distribution of disease types in our two randomization groups, thereby ensuring similar distribution of co-medication. Secondly, the assessment of VIPN in children in general is difficult. Children of younger age are not able to verbally express their complaints. Therefore, for children aged <5 years VIPN assessment often relies on parent reports [31,41], which could introduce bias. The CTCAE lacks extensive physical examination, such as manual strength testing, vibration sense or assessment of sensibility, whereas the ped-mTNS cannot be used in younger children. As a consequence, both tools might not measure the same aspects of VIPN in the same population. Nevertheless, currently they represent the best available methods for VIPN assessment in children.

Furthermore, assessors of VIPN in this study, although frequently unaware of randomization status of the patients, were not strictly blinded to the randomization status. This could have introduced bias in the VIPN results reported.

Finally, VIPN is a multifactorial phenomenon, also influenced by PK of VCR and single nucleotide polymorphisms [1]. It would be beneficial to study the impact of administration duration on VIPN while also considering these factors. Data on VCR PK and SNP’s were also collected as part of this trial, but analyses of these data was beyond the scope of this paper. Data of this trial regarding administration duration related to PK were published separately [42]. Potentially, infusion of VCR in one-hour could lead to an increased risk of extravasation, which is dangerous in VCR treatment. However, in all our patients, VCR was administered using a central venous catheter, therefore, extravasation was not a potential risk factor.

## 4. Materials and Methods 

### 4.1. Trial Design 

This study is an international, multi-center, randomized controlled trial. In total, ten pediatric oncology treatment centers across the Netherlands (four) and Belgium (six) participated in the study. Written informed consent was obtained from parents or guardians of all study participants aged 2–17 years and their children (12–17 years). The protocol and consent form were approved by the Institutional Review Board (IRB) of Amsterdam UMC, location VUmc (IRB number: 2014–268, EUDRACT number: 2014-001561-27).

The study was conducted in accordance with the Declaration of Helsinki. An independent safety committee was updated annually on study progress, incidence and nature of serious adverse events occurring during the study. This committee did not find any reason to discontinue the study.

### 4.2. Study Participants

The study population consisted of newly diagnosed pediatric oncology patients aged 2–18 years. Patients were eligible for study participation when their treatment protocol included at least four VCR administrations within six weeks. Therefore, patients with the following diagnosis and corresponding treatment protocols could be included: acute lymphoblastic leukemia (ALL) (DCOG ALL-11 protocol [29], EsPhALL protocol [43], or EORTC-58081-CLG guideline [44]), Hodgkin’s lymphoma (EuroNet-PHL-C1 protocol [45] or C2 protocol [40]), nephroblastoma (SIOP Wilms 2001 protocol [46]), and rhabdomyosarcoma (EpSSG RMS 2005 protocol [47]). Furthermore, patients with low-grade glioma (LGG) (SIOP-LGG 2004 [48] protocol) and medulloblastoma (ACNS0331 [49] protocol or ACNS0332 [50] protocol) were eligible if their presenting symptoms did not include any sensory or motor symptoms of their limbs. Patients with mental retardation or pre-existent peripheral neuropathy were excluded from participation.

### 4.3. Trial Regimen

Participating patients underwent baseline VIPN assessment to exclude pre-existing peripheral neuropathy. Then, patients were 1:1 randomized using Tenalea software (Trans European Network for Clinical Trials Services) to receive all VCR administrations through push injections or one-hour infusions. To ensure equal distribution, stratified block randomization (maximum block size: four) was used according to age group (2–10 years or 11–18 years), gender, and country. During the first year of treatment, VIPN was measured 1–3 times, depending on protocol treatment duration and number of VCR administrations (see Figure S1 for the detailed measurement schedules). In *n* = 5 patients, five measurements were done within the first year as these patient were treated according to a rather dense protocol (i.e. many VCR administrations within a relatively short period of time).

VCR was administered using a standard dose of 1.5 mg/m^2^ or 2 mg/m^2^ (maximum 2 mg), depending on treatment protocol. A push injection was administered in an injection of 10 mL 0.9% NaCl or iv bag of 50 mL 0.9% NaCl. Total administration time of a push injection was 1–5 min. One-hour infusions were administered using an iv bag of 50 mL of NaCl 0.9%.

### 4.4. Assessments of VIPN and Other Study Outcomes

The primary endpoint of the current study was VIPN measured by the CTCAE (version 4.03 [51]) (CTCAE). The items peripheral sensory neuropathy (range 0–5), peripheral motor neuropathy (range 0–5), constipation (range 0–5), and neuralgia (range 0–3) were assessed. A CTCAE score of ≥2 on at least one of the four items was defined as VIPN and ≥3 or higher as severe VIPN. Moreover, all four items were summed into a total CTCAE score (maximum 18).

Secondary endpoints of the current study included VIPN measured by the Dutch version of the ped-mTNS [31,52] and by the use of specific analgesics for neuropathic pain. The ped-mTNS is a validated instrument, including a questionnaire (sensory, functional and autonomic symptoms) and physical examination, developed to assess VIPN in children aged ≥ 5 years. Therefore, this instrument was not used in children aged < 5 years. A total ped-mTNS score of ≥5 was defined as VIPN (total scoring range of ped-mTNS: 0–32) [31]. All VIPN measurements were performed in each hospital by specifically trained physicians or physical therapists during regular hospital visits of the patient.

Data on co-medication were collected by chart-based review. Amitriptyline and gabapentin are frequently used for the treatment of neuropathic pain caused by VIPN [6]. Therefore, children using these analgesics for ≥14 days were considered to have VIPN. Furthermore, data regarding concurrent azole antifungal treatment were collected. Patients were considered to have been treated with this concurrent therapy when azoles were used during the week preceding or following VCR administration and when ≥50% of VCR administrations between two succeeding study measurements were given with concurrent azole therapy.

This paper reports on data collected during the first year of treatment.

### 4.5. Sample Size Calculation

Initial sample size calculation was based on total CTCAE scores (primary endpoint) using data of two studies [15,53] in which a mean maximum (standard deviation (SD)) CTCAE score of 2.43 (1.07) was reported. However, since our study population included children with different pediatric oncology diagnoses, we increased the expected SD to 1.3 instead of 1.07. A difference in CTCAE score of at least 1.0 was considered to be clinically relevant. This resulted in a targeted sample size of at least 70 patients (35 patients in each intervention group; α = 5%, power = 90%). To compensate for an expected drop-out rate of 25%, we aimed to include 44 patients in each group (total: 88).

### 4.6. Statistical Analyses

Descriptive data of normally and skewed distributed variables were reported as means (SD) and medians (interquartile range (IQR)), respectively. Possible associations between VCR administration method (push injection or one hour infusion) and VIPN were evaluated in multiple ways. Mean, median scores and proportions were compared using *t*-tests, Mann-Whitney U and chi-square tests, respectively. Moreover, linear mixed effects model analyses were used to assess the association between administration method and total CTCAE score as well as total ped-mTNS score, thereby correcting for the correlated observations within and between patients. Logistic generalized estimating equations (GEE) analyses were used for dichotomized CTCAE and ped-mTNS scores (i.e., having VIPN yes or no) and use of analgesics. Furthermore, the possible confounding and/or modifying effect of the covariates age, sex, cumulative VCR dose per m^2^, cancer diagnosis, concurrent azole antifungal treatment (yes/no), and self-declared ancestry was studied. When a modifying effect on the association between randomization and VIPN for one of the covariates was found (i.e., *p* value of the interaction term <0.05), results were reported for each category of this covariate separately.

All participants were included in our intention-to-treat analyses. A two-tailed *p*-value of <0.05 was considered statistically significant, analyses were performed using SPSS 26.0 (Chicago, USA) software.

## 5. Conclusions

In conclusion, our study showed that one-hour infusions of VCR are overall not associated with less VIPN compared to push administrations in pediatric cancer patients. However, when concurrent treatment with azole antifungals or similar medication affecting VCR-metabolism is necessary, iv VCR one-hour infusions seem to be beneficial over push injections, since these lead to less severe VIPN, although larger trials are needed to confirm this association.

## Figures and Tables

**Table 1:** Demographic and clinical characteristics of the participants at baseline.

	One-Hour Administration Group (*n* = 45)	Push Administration Group (*n* = 45)
Sex		
Male	26 (58)	24 (53)
Female	19 (42)	21 (47)
Age, years (mean(SD))	9.06 (5.11)	9.29 (5.25)
Age ≥ 5 years and included for ped-mTNS assessment	32 (71)	34 (76)
Disease		
Acute lymphoblastic leukemia	29 (64)	29 (64)
Hodgkin lymphoma	7 (16)	11 (24)
Nephroblastoma	6 (13)	2 (4)
Medulloblastoma	1 (2)	1 (2)
Rhabdomyosarcoma	2 (4)	0 (0)
Low-grade glioma	0 (0)	2 (4)
Ancestry ^a^		
Europe	36 (80)	37 (82)
Eastern Asia	1 (2)	0 (0)
Latin-America (including Caribbean)	1 (2)	2 (4)
Middle-East (including Northern Africa)	3 (7)	3 (7)
Sub-Saharan Africa	1 (2)	0 (0)
Combination	2 (4)	3 (7)
Missing	1 (2)	0 (0)
No. (%) of patients needing VCR dose reductions or omissions	1 (3)	0 (0)
No. (%) of patients using analgesics for neuropathic pain	9 (20)	11 (24)
Relapse (No. (%))	2 (4)	1 (2)
Death (No. (%))	3 (7)	1 (2)

Values represent the number (%) of participants, unless indicated otherwise. SD: standard deviation; ped-mTNS: pediatric modified Total Neuropathy Score; VCR: vincristine, ^a^: Ancestry was self- or parent-reported; participants could only be included in one category.

**Table 2:** Incidence of vincristine induced peripheral neuropathy of participants in both randomization groups.

	One-Hour (*n* = 45) *n* (%) *	Push (*n* = 45) *n* (%) *	*p* Value
VIPN based on CTCAE	21 (46.7)	23 (51.1)	0.67
Severe VIPN based on CTCAE	3 (6.7)	6 (13.3)	0.49
VIPN based on ped-mTNS	27 (84.4) **	28 (82.4) **	0.83
VIPN pain medication	9 (20.0)	11 (24.4)	0.61
VIPN based on CTCAE item constipation	3 (6.7)	8 (17.8)	0.20
VIPN based on CTCAE item peripheral sensory neuropathy	5 (11.1)	6 (13.3)	0.75
VIPN based on CTCAE item peripheral motor neuropathy	17 (37.8)	15 (33.3)	0.66
VIPN based on CTCAE item neuralgia	6 (13.3)	12 (26.7)	0.11
CTCAE score (median [IQR])	2.00 [1.00–3.00]	1.50 [0.00–3.00]	0.43
ped-mTNS score (median [IQR])	6.00 [3.75–10.00] **	5.50 [2.00–9.00] **	0.19
VIPN outcomes without concurrent azole antifungals	**One-hour (*n* = 35)**	**Push (*n* = 36)**	
CTCAE score without concurrent azole treatment (median [IQR])	2.00 [1.00–2.75]	1.00 [0.00–3.00]	0.20
ped-mTNS score without concurrent azole treatment (median [IQR])	5.50 [3.25–9.00] ***	5.00 [2.00–8.00] ***	0.09
VIPN outcomes with concurrent azole antifungals	**One-hour (*n* = 10)**	**Push (*n* = 9)**	
CTCAE score with concurrent azole treatment (median [IQR])	2.00 [1.00–3.00]	3.00 [1.00–6.00]	0.21
ped-mTNS score with concurrent azole treatment (median [IQR])	8.50 [3.75–11.50] ****	10.00 [7.00–13.00] ****	0.39

VIPN: vincristine induced peripheral neuropathy, CTCAE: common terminology criteria of adverse events, ped-mTNS: pediatric-modified Total Neuropathy Score, IQR: interquartile range * All scores indicate *n* (%) unless indicated otherwise ** Total group was *n* = 32 in the iv one-hour group and *n* = 34 in the iv push group since ped-mTNS assessment was only done in children aged ≥5 years, *** Total group was *n* = 23 in the iv one-hour group and *n* = 27 in the iv push group, **** Total group was *n* = 9 in the iv one-hour group and *n* = 7 in the iv push group.

**Table 3:** The effect of VCR administration method (push administration versus one-hour administration) on vincristine-induced peripheral neuropathy.

	Total Group (*n* = 90)		Subgroup of Participants without Concurrent Azole Treatment (*n* = 71)	Subgroup of Participants with Concurrent Azole Treatment (*n* = 19)		
	β/OR (95% CI)	*p*-Value	β/OR (95% CI)	*p*-Value	β/OR (95% CI)	*p*-Value
CTCAE ^a^ Total score during treatment	−0.29 (−0.89 to 0.31)	0.34	−0.12 (−0.73 to 0.49)	0.69	−1.58 (−3.11 to −0.05)	0.04
Ped-mTNS ^a^ Total score during treatment	−0.25 (−1.95 to 1.45)	0.77	0.03 (−1.78 to 1.84)	0.97	−1.80 (−5.33 to 1.72)	0.31
Participants with VIPN ^a,b^ Based on CTCAE Based on ped-mTNS Based on analgesics use	1.21 (0.43 to 1.46) 1.12 (0.58 to 2.16) 0.76 (0.30 to 1.94)	0.45 0.74 0.57	0.93 (0.49 to 1.79) 1.33 (0.63 to 2.80) 0.67 (0.21 to 2.18)	0.84 0.46 0.51	0.26 (0.04 to 1.67) 0.22 (0.02 to 2.76) 0.86 (0.17 to 4.43)	0.16 0.24 0.86

OR: odds ratio; 95% CI: 95% confidence interval; Common Terminology Criteria of Adverse Events; ped-mTNS: pediatric modified Total Neuropathy Score; VIPN: vincristine-induced peripheral neuropathy; ^a^: Push administration group served as reference group, ^b^ No VIPN group served as reference group. Results regarding CTCAE were the primary endpoint in this study, results regarding ped-mTNS and analgesics were secondary outcomes.

## Supplementary Materials

The following are available online at https://www.mdpi.com/2072-6694/12/12/3745/s1, Table S1: Baseline VIPN scores of patients of the intention-to-treat group for both randomization groups, Table S2: The effect of VCR administration method (push administration versus one-hour administration) on vincristine-induced peripheral neuropathy in the intention-to-treat analysis additionally corrected for age, sex, VCR dose per m^2^, cancer diagnosis and ethnicity, Figure S1: Measurement schedule per treatment protocol, Supplementary Study protocol: Vincristine-Induced Neuropathy in children with CAncer (the VINCA study) version 5.3

## References

[1] van de Velde M.E., Kaspers G.L., Abbink F.C.H., Wilhelm A.J., Ket J.C.F., van den Berg M.H. (2017). Vincristine-induced peripheral neuropathy in children with cancer: A systematic review. Crit. Rev. Oncol. Hematol..

[2] Gidding C.E., Kellie S.J., Kamps W.A., De Graaf S.S. (1999). Vincristine revisited. Crit. Rev. Oncol. Hematol..

[3] Coccia P.F., Altman J., Bhatia S., Borinstein S.C., Flynn J., George S., Goldsby R., Hayashi R., Huang M.S., Johnson R.H. (2012). Adolescent and young adult oncology clinical practice guidelines in oncology. JNCCN J. Natl. Compr. Cancer Netw..

[4] Stryckmans P.A., Lurie P.M., Manaster J., Vamecq G. (1973). Mode of action of chemotherapy in vivo on human acute leukemia--II. Vincristine. Eur. J. Cancer.

[5] Dennison J.B., Jones D.R., Renbarger J.L., Hall S.D. (2007). Effect of CYP3A5 expression on vincristine metabolism with human liver microsomes. J. Pharmacol. Exp. Ther..

[6] Anghelescu D.L., Faughnan L.G., Jeha S., Relling M.V., Hinds P.S., Sandlund J.T., Cheng C., Pei D., Hankins G., Pauley J.L. (2011). Neuropathic pain during treatment for childhood acute lymphoblastic leukemia. Pediatr. Blood Cancer.

[7] Gutierrez-Camino A., Martin-Guerrero I., Lopez-Lopez E., Echebarria-Barona A., Zabalza I., Ruiz I., Guerra-Merino I., Garcia-Orad A. (2016). Lack of association of the CEP72 rs924607 TT genotype with vincristine-related peripheral neuropathy during the early phase of pediatric acute lymphoblastic leukemia treatment in a Spanish population. Pharmacogenet. Genom..

[8] Gilchrist L.S., Marais L., Tanner L. (2014). Comparison of two chemotherapy-induced peripheral neuropathy measurement approaches in children. Supportive Care Cancer.

[9] Gutierrez-Gutierrez G., Sereno M., Miralles A., Casado-Saenz E., Gutierrez-Rivas E. (2010). Chemotherapy-induced peripheral neuropathy: Clinical features, diagnosis, prevention and treatment strategies. Clin. Transl. Oncol.

[10] Gomber S., Dewan P., Chhonker D. (2010). Vincristine induced neurotoxicity in cancer patients. Indian J. Pediatrics.

[11] Windebank A.J., Grisold W. (2008). Chemotherapy-induced neuropathy. J. Peripher. Nerv. Syst.

[12] Beijers A.J., Jongen J.L., Vreugdenhil G. (2012). Chemotherapy-induced neurotoxicity: the value of neuroprotective strategies. Neth. J. Med..

[13] Rosca L., Robert-Boire V., Delisle J.F., Samson Y., Perreault S. (2018). Carboplatin and vincristine neurotoxicity in the treatment of pediatric low-grade gliomas. Pediatr. Blood Cancer.

[14] Diouf B., Crews K.R., Lew G., Pei D., Cheng C., Bao J., Zheng J.J., Yang W., Fan Y., Wheeler H.E. (2015). Association of an inherited genetic variant with vincristine-related peripheral neuropathy in children with acute lymphoblastic leukemia. JAMA J. Am. Med Assoc..

[15] Egbelakin A., Ferguson M.J., MacGill E.A., Lehmann A.S., Topletz A.R., Quinney S.K., Li L., McCammack K.C., Hall S.D., Renbarger J.L. (2011). Increased risk of vincristine neurotoxicity associated with low CYP3A5 expression genotype in children with acute lymphoblastic leukemia. Pediatric Blood Cancer.

[16] Kishi S., Cheng C., French D., Pei D., Das S., Cook E.H., Hijiya N., Rizzari C., Rosner G.L., Frudakis T. (2007). Ancestry and pharmacogenetics of antileukemic drug toxicity. Blood.

[17] Ceppi F., Langlois-Pelletier C., Gagné V., Rousseau J., Iolino C., Orenzo S.D., Evin K.M., Ijov D., Allan S.E., Ilverman L.B. (2014). Polymorphisms of the vincristine pathway and response to treatment in children with childhood acute lymphoblastic leukemia. Pharmacogenomics.

[18] Abaji R., Ceppi F., Patel S., Gagne V., Xu C.J., Spinella J.F., Colombini A., Parasole R., Buldini B., Basso G. (2018). Genetic risk factors for VIPN in childhood acute lymphoblastic leukemia patients identified using whole-exome sequencing. Pharmacogenomics.

[19] Lopez-Lopez E., Gutierrez-Camino A., Astigarraga I., Navajas A., Echebarria-Barona A., Garcia-Miguel P., Garcia de Andoin N., Lobo C., Guerra-Merino I., Martin-Guerrero I. (2016). Vincristine pharmacokinetics pathway and neurotoxicity during early phases of treatment in pediatric acute lymphoblastic leukemia. Pharmacogenomics.

[20] Aplenc R., Glatfelter W., Han P., Rappaport E., La M., Cnaan A., Blackwood M.A., Lange B., Rebbeck T. (2003). CYP3A genotypes and treatment response in paediatric acute lymphoblastic leukaemia. Br. J. Haematol..

[21] Renbarger J.L., McCammack K.C., Rouse C.E., Hall S.D. (2008). Effect of race on vincristine-associated neurotoxicity in pediatric acute lymphoblastic leukemia patients. Pediatr. Blood Cancer.

[22] Van Schie R.M., Bruggemann R.J., Hoogerbrugge P.M., Te Loo D.M. (2011). Effect of azole antifungal therapy on vincristine toxicity in childhood acute lymphoblastic leukaemia. J. Antimicrob. Chemother..

[23] Moriyama B., Henning S.A., Leung J., Falade-Nwulia O., Jarosinski P., Penzak S.R., Walsh T.J. (2012). Adverse interactions between antifungal azoles and vincristine: review and analysis of cases. Mycoses.

[24] Baxter C.G., Marshall A., Roberts M., Felton T.W., Denning D.W. (2011). Peripheral neuropathy in patients on long-term triazole antifungal therapy. J. Antimicrob. Chemother..

[25] Science M., Robinson P.D., MacDonald T., Rassekh S.R., Dupuis L.L., Sung L. (2014). Guideline for primary antifungal prophylaxis for pediatric patients with cancer or hematopoietic stem cell transplant recipients. Pediatr. Blood Cancer.

[26] Kellie S.J., Koopmans P., Earl J., Nath C., Roebuck D., Uges D.R.A., De Graaf S.S.N. (2004). Increasing the dosage of vincristine: A clinical and pharmacokinetic study of continuous-infusion vincristine in children with central nervous system tumors. Cancer.

[27] Pinkerton C.R., McDermott B., Philip T., Biron P., Ardiet C., Vandenberg H., Brunat-Mentigny M. (1988). Continuous vincristine infusion as part of a high dose chemoradiotherapy regimen: Drug kinetics and toxicity. Cancer Chemother. Pharmacol..

[28] Gidding C.E., Fock J.M., Begeer J.H., Koopmans P., Meeuwsen-de Boer G.J., Kamps W.A., Uges D.R.A., De Graaf S.S.N. Vincristine disposition and neurotoxicity in children. Proceedings of the Annual Meeting-American Society of Clinical Oncology.

[29] DCOG Protocol ALL-11 (2013): Treatment Study Protocol of the Dutch Childhood Oncology Group for Children and Adolescents (1–19 year) with Newly Diagnosed Acute Lymphoblastic Leukemia. Version 4.0. https://www.skion.nl/workspace/uploads/Onderzoeksprotocol-ALL11-version-4-3-september-2014.pdf.

[30] Varedi M., Lu L., Howell C.R., Partin R.E., Hudson M.M., Pui C.H., Krull K.R., Robison L.L., Ness K.K., McKenna R.F. (2018). Peripheral Neuropathy, Sensory Processing, and Balance in Survivors of Acute Lymphoblastic Leukemia. J. Clin. Oncol..

[31] Gilchrist L.S., Tanner L. (2013). The pediatric-modified total neuropathy score: A reliable and valid measure of chemotherapy-induced peripheral neuropathy in children with non-CNS cancers. Supportive Care Cancer.

[32] Smolik S., Arland L., Hensley M.A., Schissel D., Shepperd B., Thomas K., Rodgers C. (2018). Assessment Tools for Peripheral Neuropathy in Pediatric Oncology: A Systematic Review From the Children’s Oncology Group. J. Pediatr. Oncol. Nurs..

[33] Leverger G., Timsit J.F., Milpied N., Gachot B. (2019). Use of Micafungin for the Prevention and Treatment of Invasive Fungal Infections in Everyday Pediatric Care in France: Results of the MYRIADE Study. Pediatr. Infect Dis. J..

[34] Bochennek K., Balan A., Muller-Scholden L., Becker M., Farowski F., Muller C., Groll A.H., Lehrnbecher T. (2015). Micafungin twice weekly as antifungal prophylaxis in paediatric patients at high risk for invasive fungal disease. J. Antimicrob Chemother..

[35] Lee C.H., Lin J.C., Ho C.L., Sun M., Yen W.T., Lin C. (2017). Efficacy and safety of micafungin versus extensive azoles in the prevention and treatment of invasive fungal infections for neutropenia patients with hematological malignancies: A meta-analysis of randomized controlled trials. PLoS ONE.

[36] Fisher B.T., Zaoutis T., Dvorak C.C., Nieder M., Zerr D., Wingard J.R., Callahan C., Villaluna D., Chen L., Dang H. (2019). Effect of Caspofungin vs Fluconazole Prophylaxis on Invasive Fungal Disease among Children and Young Adults with Acute Myeloid Leukemia: A Randomized Clinical Trial. JAMA.

[37] Papachristou S., Iosifidis E., Roilides E. (2019). Invasive Aspergillosis in Pediatric Leukemia Patients: Prevention and Treatment. J. Fungi..

[38] Saad A.H., DePestel D.D., Carver P.L. (2006). Factors influencing the magnitude and clinical significance of drug interactions between azole antifungals and select immunosuppressants. Pharmacotherapy.

[39] Pana Z.D., Roilides E. (2011). Risk of azole-enhanced vincristine neurotoxicity in pediatric patients with hematological malignancies: old problem—New dilemma. Pediatr. Blood Cancer.

[40] The EuroNet-PHL-C2 Protocol (2016) Second International Inter-Group Study for Classical Hodgkin’s Lymphoma in Children and Adolescents. Version 3.0. https://clinicaltrials.gov/ct2/show/NCT02684708.

[41] Lavoie Smith E.M., Li L., Hutchinson R.J., Ho R., Burnette W.B., Wells E., Bridges C., Renbarger J. (2013). Measuring vincristine-induced peripheral neuropathy in children with acute lymphoblastic leukemia. Cancer Nurs..

[42] Van de Velde M.E., Panetta J.C., Wilhelm A.J., van den Berg M.H., van der Sluis I.M., van den Bos C., Abbink F.C.H., van den Heuvel-Eibrink M.M., Segers H., Chantrain C. (2020). Population Pharmacokinetics of Vincristine Related to Infusion Duration and Peripheral Neuropathy in Pediatric Oncology Patients. Cancers.

[43] EsPhALL (2015): An Open-Label Study to Evaluate the Safety and Efficacy of IMATINIB with Chemotherapy in Pediatric Patients with Ph+/BCR-ABL+ Acute Lymphoblastic Leukemia (Ph+ALL). https://www.skion.nl/workspace/uploads/EsPhALL_Amendment-2_01102015_final_1.pdf.

[44] EORTC-58081-CLG Translational Research-Observational Study for Identification of New Possible Prognostic Factors and Future Therapeutic Targets in Children with Acute Lymphoblastic Leukaemia (ALL). https://www.eortc.org/research_field/clinical-detail/58081/.

[45] The EuroNet-PHL-C1 Protocol (2012) First International Inter-Group Study for Classical Hodgkin’s Lymphoma in Children and Adolescents. https://www.skion.nl/workspace/uploads/euronet-phl-c1_workingcopy_inkl_amendm06_mw_2012-11-14_0.pdf.

[46] SIOP Wilms (2001): Chemotherapy Before and After Surgery in Treating Children With Wilm’s tumor. https://clinicaltrials.gov/ct2/show/NCT00047138.

[47] The EpSSG Protocol (2012) A Protocol for Non Metastatic Rhabdomyosarcoma. Version 1.3. https://www.skion.nl/workspace/uploads/Protocol-EpSSG-RMS-2005-1-3-May-2012_1.pdf.

[48] SIOP LGG 2004: Cooperative Multicenter Study for Children and Adolescents with Low Grade Glioma. https://www.kinderkrebsinfo.de/sites/kinderkrebsinfo/content/e1676/e9032/e68518/e5400/download7688/Master-ProtokollGPOHVersionI,April2004_Druck_KLEIN_ger.pdf.

[49] ACNS0331 (2004): A Study Evaluating Limited Target Volume Boost Irradiation and Reduced Dose Craniospinal Radiotherapy (18.00 Gy) and Chemotherapy in Children with Newly Diagnosed Standard Risk Medulloblastoma: A Phase III Double Randomized Trial. https://www.childrensoncologygroup.org/acns0331.

[50] ACNS0332 (2007): Efficacy of Carboplatin Administered Concomitantly with Radiation and Isotretinoin as a Pro-Apoptotic Agent in Other than Average Risk Medulloblastoma/PNET Patients. https://clinicaltrials.gov/ProvidedDocs/27/NCT00392327/Prot_SAP_000.pdf.

[51] National Institutes of Health, N.C.I. (2010) Common Terminology Criteria for Adverse Events (CTCAE) Version 4.03. https://evs.nci.nih.gov/ftp1/CTCAE/CTCAE_4.03/CTCAE_4.03_2010-06-14_QuickReference_8.5x11.pdf.

[52] Schouten S.M., van de Velde M.E., Kaspers G.J.L., Mokkink L.B., van der Sluis I.M., van den Bos C., Hartman A., Abbink F.C.H., van den Berg M.H. (2019). Measuring vincristine-induced peripheral neuropathy in children with cancer: validation of the Dutch pediatric-modified Total Neuropathy Score. Support Care Cancer.

[53] Purser M.J., Johnston D.L., McMillan H.J. (2014). Chemotherapy-induced peripheral neuropathy among paediatric oncology patients. Can. J. Neurol. Sci..

