# Correction: van de Velde et al. Vincristine-Induced Peripheral Neuropathy in Pediatric Oncology: A Randomized Controlled Trial Comparing Push Injections with One-Hour Infusions (The VINCA Trial). *Cancers* 2020, *12*, 3745

**DOI:** 10.3390/cancers15051359

**Published:** 2023-02-21

**Authors:** Mirjam Esther van de Velde, Gertjan J. L. Kaspers, Floor C. H. Abbink, Jos W. R. Twisk, Inge M. van der Sluis, Cor van den Bos, Marry M. van den Heuvel-Eibrink, Heidi Segers, Christophe Chantrain, Jutte van der Werff ten Bosch, Leen Willems, Marleen H. van den Berg

**Affiliations:** 1 Emma Children’s Hospital, Amsterdam UMC, Vrije Universiteit Amsterdam, Pediatric Oncology, 1081 HV Amsterdam, The Netherlands; 2 Princess Máxima Center for Pediatric Oncology, 3584 CS Utrecht, The Netherlands; 3 Emma Children’s Hospital, Amsterdam UMC, Amsterdam Medical Center, Pediatric Oncology, 1105 AZ Amsterdam, The Netherlands; 4 Department of Epidemiology and Biostatistics, Amsterdam UMC, Vrije Universiteit Amsterdam, 1081 HV Amsterdam, The Netherlands; 5 Department of Pediatric Oncology, Erasmus Medical Center Rotterdam/Sophia Children’s Hospital, 3000 CB Rotterdam, The Netherlands; 6 Department of Pediatric Hemato-Oncology, University Hospitals Leuven, 3000 Leuven, Belgium; 7 Department of Pediatrics, Clinique du MontLégia, CHC, 4000 Liège, Belgium; 8 Department of Pediatric Onco-Hematology, Universitair Ziekenhuis Brussel, 1090 Brussels, Belgium; 9 Department of Paediatric Haematology-Oncology and Stem Cell Transplantation, Ghent University Hospital, 9000 Ghent, Belgium

## Error in Table

In the original publication, there was a mistake in **Table 1** as published [1]. The number of patients with death was swapped between the one-hour administration group and the push administration group. The corrected Table 1 appears below. The authors state that the scientific conclusions are unaffected. This correction was approved by the Academic Editor. The original publication has also been updated.

## Figures and Tables

**Table 1:** Demographic and clinical characteristics of the participants at baseline.

	One-Hour Administration Group (*n* = 45)	Push Administration Group (*n* = 45)
Sex		
Male	26 (58)	24 (53)
Female	19 (42)	21 (47)
Age, years (mean(SD))	9.06 (5.11)	9.29 (5.25)
Age ≥ 5 years and included for ped-mTNS assessment	32 (71)	34 (76)
Disease		
Acute lymphoblastic leukemia	29 (64)	29 (64)
Hodgkin lymphoma	7 (16)	11 (24)
Nephroblastoma	6 (13)	2 (4)
Medulloblastoma	1 (2)	1 (2)
Rhabdomyosarcoma	2 (4)	0 (0)
Low-grade glioma	0 (0)	2 (4)
Ancestry ^a^		
Europe	36 (80)	37 (82)
Eastern Asia	1 (2)	0 (0)
Latin-America (including Caribbean)	1 (2)	2 (4)
Middle-East (including Northern Africa)	3 (7)	3 (7)
Sub-Saharan Africa	1 (2)	0 (0)
Combination	2 (4)	3 (7)
Missing	1 (2)	0 (0)
No. (%) of patients needing VCR dose reductions or omissions	1 (3)	0 (0)
No. (%) of patients using analgesics for neuropathic pain	9 (20)	11 (24)
Relapse (No. (%))	2 (4)	1 (2)
Death (No. (%))	3 (7)	1 (2)

Values represent the number (%) of participants, unless indicated otherwise. SD: standard deviation; ped-mTNS: pediatric modified Total Neuropathy Score; VCR: vincristine, ^a^: Ancestry was self- or parent-reported; participants could only be included in one category.
