# Supplementary material for: Vincristine-Induced Peripheral Neuropathy in Pediatric Oncology: A Randomized Controlled Trial Comparing Push Injections with One-Hour Infusions (The VINCA Trial)
Source: Cancers (Basel). 2020 Dec 12;12(12):3745. doi: 10.3390/cancers12123745 (PMC7764775; doi:10.3390/cancers12123745)
Supplement: Supplementary file 1 [file cancers-12-03745-s001.pdf]

Supplementary Materials

Vincristine-Induced Peripheral Neuropathy in Pediatric Oncology: A Randomized Controlled Trial Comparing Push Injections with One-Hour Infusions (The VINCA Trial)

Mirjam Esther van de Velde, Gertjan J.L. Kaspers, Floor C.H. Abbink, Jos W.R. Twisk, Inge M. van der Sluis, Cor van den Bos, Marry M. van den Heuvel–Eibrink, Heidi Segers, Christophe Chantrain, Jutte van der Werff Ten Bosch, Leen Willems and Marleen H. van den Berg

Table S1. Baseline vincristine induced peripheral neuropathy scores of patients of the intention-to-treat group for both randomization groups.

	One-hour administration group (n=45)		Push administration group (n=45)	
	**No. of participants**	Median (IQR)	No. of participants	Median (IQR)
**CTCAE**				
**Total score**	45	0.00 [0.00 – 1.00]	45	0.00 [0.00 – 0.00]
**Item sensory peripheral sensory neuropathy**	44	0.00 [0.00 – 0.00]	45	0.00 [0.00 – 0.00]
**Item peripheral motor neuropathy**	43	0.00 [0.00 – 0.00]	45	0.00 [0.00 – 0.00]
**Item constipation**	45	0.00 [0.00 – 1.00]	45	0.00 [0.00 – 0.00]
**Item neuralgia**	44	0.00 [0.00 – 0.00]	45	0.00 [0.00 – 0.00]
**ped-mTNS**				
**Total score**	29	1.00 [0.00 – 2.50]	34	1.00 [0.00 – 2.00]

No.: number; IQR: interquartile range, Common Toxicity Criteria of Adverse Events; ped-mTNS: pediatric modified Total Neuropathy Score.

Table S2. The effect of VCR administration method (push administration versus one-hour administration) on vincristine-induced peripheral neuropathy in the intention-to-treat analysis additionally corrected for age, sex, VCR dose per m², cancer diagnosis and ethnicity.

	**Total group (n=90)**		**Subgroup of participants without concurrent azole treatment (n=71)**		**Subgroup of participants with concurrent azole treatment (n=19)**	
	**β/ OR* (95% CI)**	**p-value**	**β/ OR* (95% CI)**	**p-value**	**β/ OR* (95% CI)**	**p-value**
**CTCAE[a] Total score during treatment**	-0.04 (-0.61 to 0.54)	0.90	0.20 (-0.39 to 0.79)	0.50	-1.72 (-3.19 to -0.25)	0.02
**Ped-mTNS[a] Total score during treatment**	0.13 (-1.50 to 1.76)	0.87	0.76 (-1.00 to 2.52)	0.39	-2.87 (-6.27 to 0.53)	0.10
**Participants with VIPN[a,b] Based on CTCAE**	0.94 (0.51 to 1.74)	0.85	1.18 (0.61 to 2.26) 1.78 (0.74 to 4.29)	0.63	0.22 (0.03 to 1.78) 0.17 (0.02 to 1.60)	0.16

	1.39 (0.63 to 3.08)	0.42	1.33 (0.42 to 4.25)	0.20	0.70 (0.09 to 5.39)	0.12
**Based on ped-mTNS total score**	1.13 (0.39 to 3.23)	0.82		0.63		0.70
**Based on analgesics use**						

OR: odds ratio; 95% CI: 95% confidence interval; Common Toxicity Criteria of Adverse Events; ped-mTNS: pediatric modified Total Neuropathy Score; VIPN: vincristine-induced peripheral neuropathy; [a] Push administration group served as reference group, [b]No VIPN served as reference group.

Figure S1. Measurement schedule per treatment protocol.

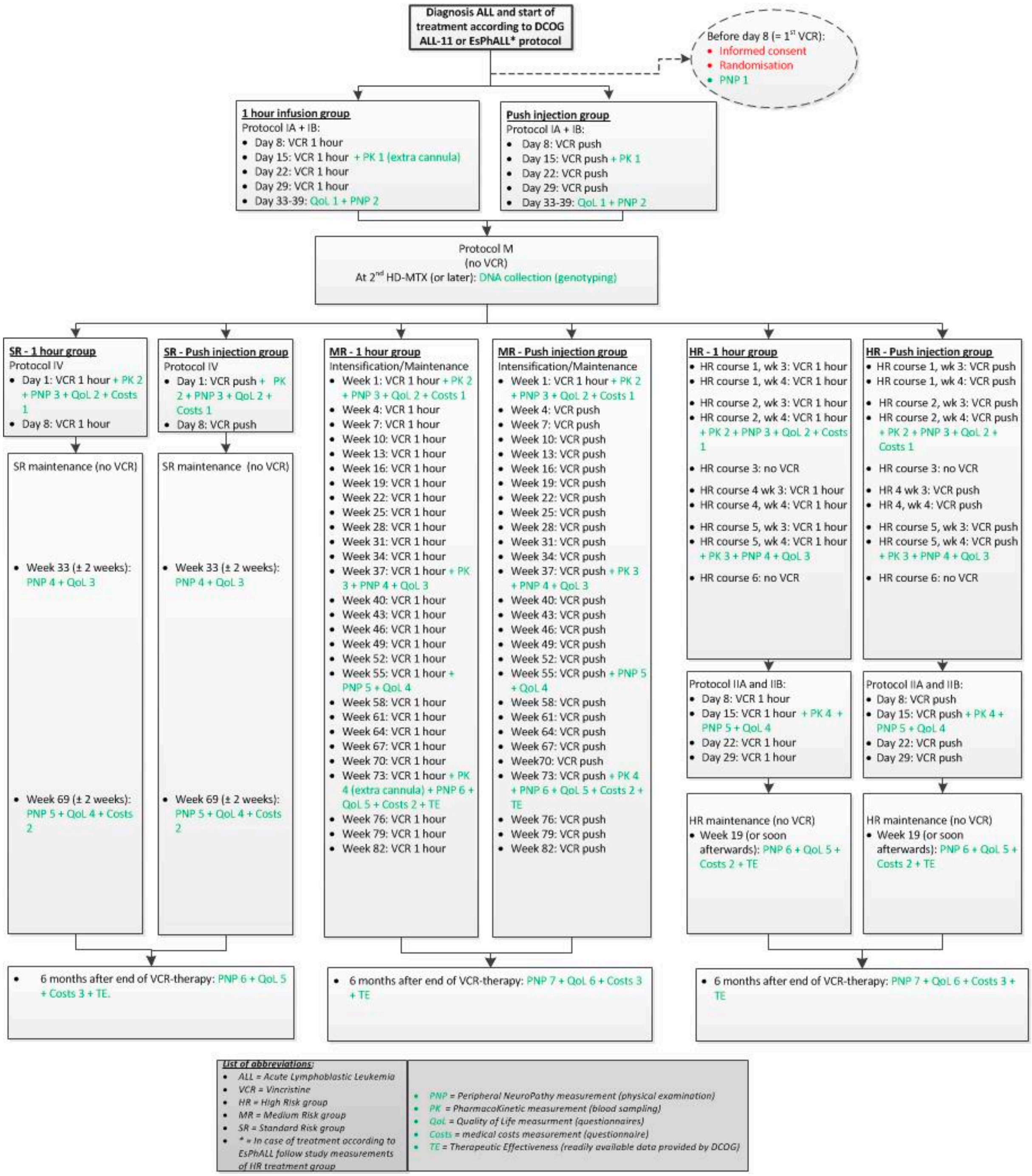

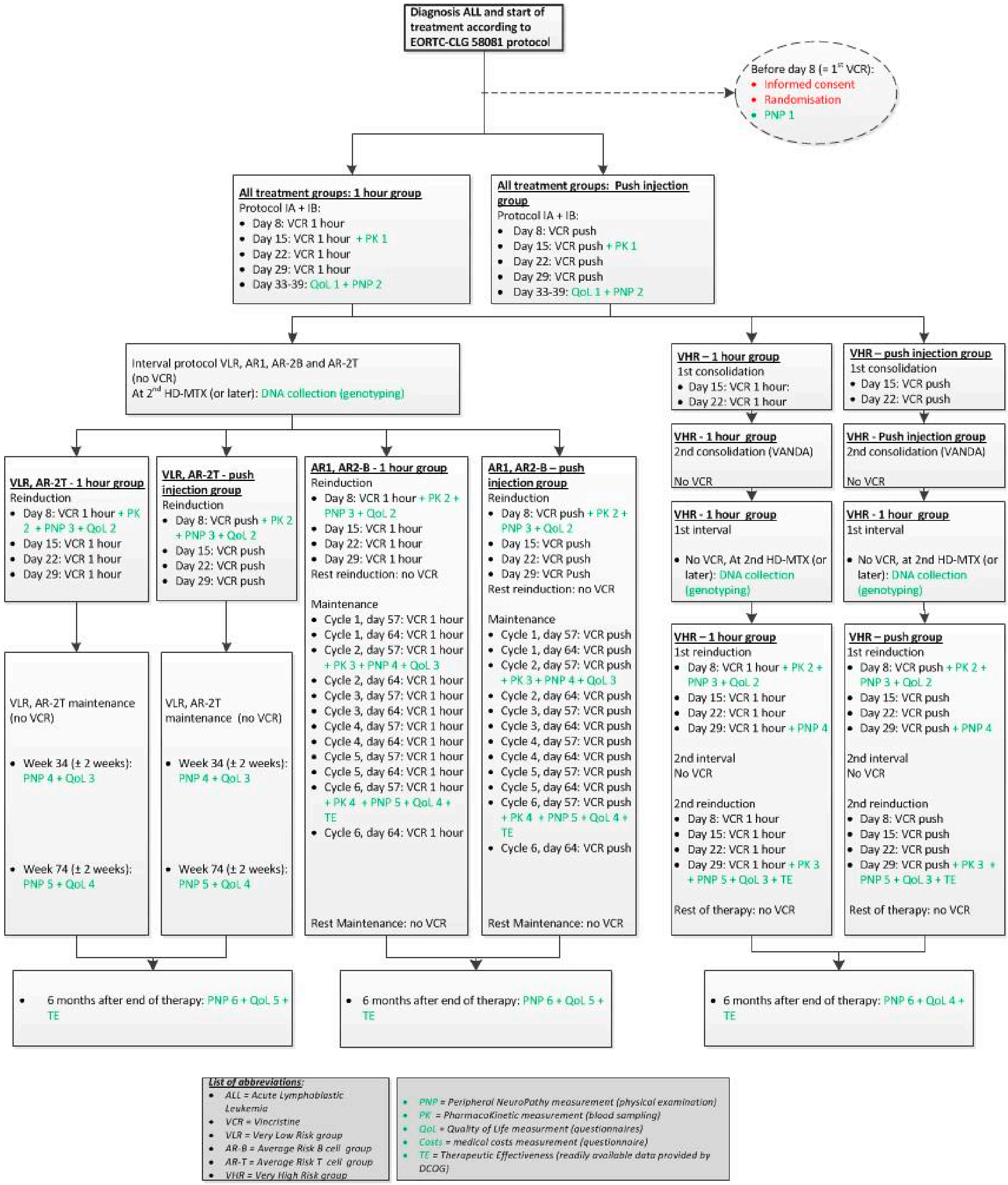
Diagnosis ALL and start of treatment according to EORTC-CLG 58081 protocol
Before day 8 (= 1st VCR):
• Informed consent
• Randomisation
• PNP 1
All treatment groups: 1 hour group
Protocol IA + IB:
• Day 8: VCR 1 hour
• Day 15: VCR 1 hour + PK 1
• Day 22: VCR 1 hour
• Day 29: VCR 1 hour
• Day 33-39: QoL 1 + PNP 2
All treatment groups: Push injection group
Protocol IA + IB:
• Day 8: VCR push
• Day 15: VCR push + PK 1
• Day 22: VCR push
• Day 29: VCR push
• Day 33-39: QoL 1 + PNP 2
Interval protocol VLR, AR1, AR-2B and AR-2T (no VCR)
At 2nd HD-MTX (or later): DNA collection (genotyping)
VHR – 1 hour group
1st consolidation
• Day 15: VCR 1 hour;
• Day 22: VCR 1 hour
VHR – push injection group
1st consolidation
• Day 15: VCR push
• Day 22: VCR push
VHR - 1 hour group
2nd consolidation (VANDA)
No VCR
VHR - Push injection group
2nd consolidation (VANDA)
No VCR
VHR - 1 hour group
1st interval
• No VCR, At 2nd HD-MTX (or later): DNA collection (genotyping)
VHR - 1 hour group
1st interval
• No VCR, at 2nd HD-MTX (or later): DNA collection (genotyping)
VLR, AR-2T - 1 hour group
Reinduction
• Day 8: VCR 1 hour + PK 2 + PNP 3 + QoL 2
• Day 15: VCR 1 hour
• Day 22: VCR 1 hour
• Day 29: VCR 1 hour
VLR, AR-2T - push injection group
Reinduction
• Day 8: VCR push + PK 2 + PNP 3 + QoL 2
• Day 15: VCR push
• Day 22: VCR push
• Day 29: VCR push
AR1, AR2-B - 1 hour group
Reinduction
• Day 8: VCR 1 hour + PK 2 + PNP 3 + QoL 2
• Day 15: VCR 1 hour
• Day 22: VCR 1 hour
• Day 29: VCR 1 hour
Rest reinduction: no VCR
Maintenance
• Cycle 1, day 57: VCR 1 hour
• Cycle 1, day 64: VCR 1 hour
• Cycle 2, day 57: VCR 1 hour + PK 3 + PNP 4 + QoL 3
• Cycle 2, day 64: VCR 1 hour
• Cycle 3, day 57: VCR 1 hour
• Cycle 3, day 64: VCR 1 hour
• Cycle 4, day 57: VCR 1 hour
• Cycle 4, day 64: VCR 1 hour
• Cycle 5, day 57: VCR 1 hour
• Cycle 5, day 64: VCR 1 hour
• Cycle 6, day 57: VCR 1 hour + PK 4 + PNP 5 + QoL 4 + TE
• Cycle 6, day 64: VCR 1 hour
Rest Maintenance: no VCR
AR1, AR2-B – push injection group
Reinduction
• Day 8: VCR push + PK 2 + PNP 3 + QoL 2
• Day 15: VCR push
• Day 22: VCR push
• Day 29: VCR Push
Rest reinduction: no VCR
Maintenance
• Cycle 1, day 57: VCR push
• Cycle 1, day 64: VCR push
• Cycle 2, day 57: VCR push + PK 3 + PNP 4 + QoL 3
• Cycle 2, day 64: VCR push
• Cycle 3, day 57: VCR push
• Cycle 3, day 64: VCR push
• Cycle 4, day 57: VCR push
• Cycle 4, day 64: VCR push
• Cycle 5, day 57: VCR push
• Cycle 5, day 64: VCR push
• Cycle 6, day 57: VCR push + PK 4 + PNP 5 + QoL 4 + TE
• Cycle 6, day 64: VCR push
Rest Maintenance: no VCR
VHR – 1 hour group
1st reinduction
• Day 8: VCR 1 hour + PK 2 + PNP 3 + QoL 2
• Day 15: VCR 1 hour
• Day 22: VCR 1 hour
• Day 29: VCR 1 hour + PNP 4
2nd interval
No VCR
2nd reinduction
• Day 8: VCR 1 hour
• Day 15: VCR 1 hour
• Day 22: VCR 1 hour
• Day 29: VCR 1 hour + PK 3 + PNP 5 + QoL 3 + TE
Rest of therapy: no VCR
VHR – push group
1st reinduction
• Day 8: VCR push + PK 2 + PNP 3 + QoL 2
• Day 15: VCR push
• Day 22: VCR push
• Day 29: VCR push + PNP 4
2nd interval
No VCR
2nd reinduction
• Day 8: VCR push
• Day 15: VCR push
• Day 22: VCR push
• Day 29: VCR push + PK 3 + PNP 5 + QoL 3 + TE
Rest of therapy: no VCR
VLR, AR-2T maintenance (no VCR)
• Week 34 (± 2 weeks): PNP 4 + QoL 3
• Week 74 (± 2 weeks): PNP 5 + QoL 4
VLR, AR-2T maintenance (no VCR)
• Week 34 (± 2 weeks): PNP 4 + QoL 3
• Week 74 (± 2 weeks): PNP 5 + QoL 4
• 6 months after end of therapy: PNP 6 + QoL 5 + TE
• 6 months after end of therapy: PNP 6 + QoL 5 + TE
• 6 months after end of therapy: PNP 6 + QoL 4 + TE
List of abbreviations:
• ALL = Acute Lymphoblastic Leukemia
• VCR = Vincristine
• VLR = Very Low Risk group
• AR-B = Average Risk B cell group
• AR-T = Average Risk T cell group
• VHR = Very High Risk group
• PNP = Peripheral NeuroPathy measurement (physical examination)
• PK = PharmacoKinetic measurement (blood sampling)
• QoL = Quality of Life measurment (questionnaires)
• Costs = medical costs measurement (questionnaire)
• TE = Therapeutic Effectiveness (readily available data provided by DCOG)

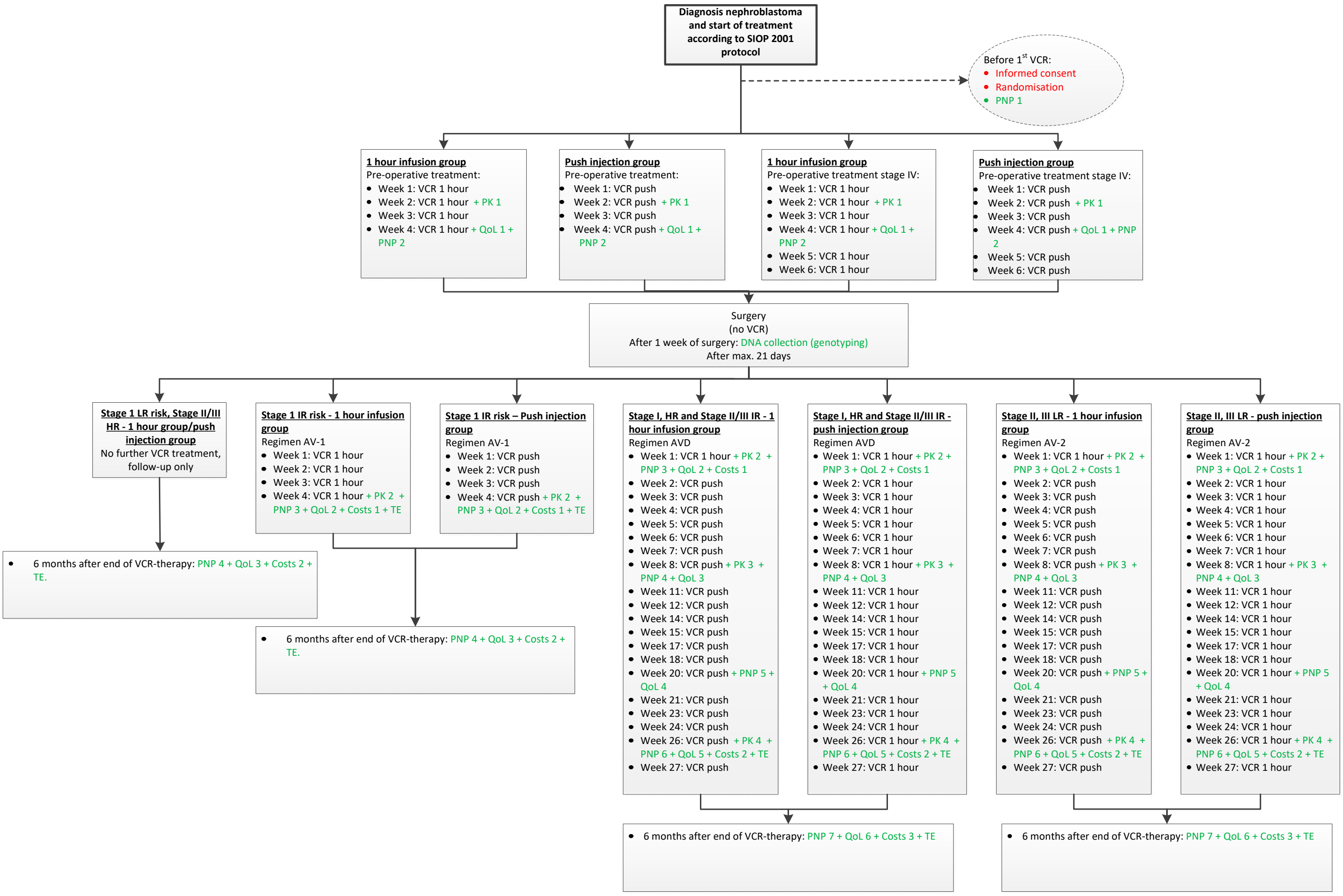

List of abbreviations:

- *SIOP = International Society of Paediatric Oncology*
- *VCR = Vincristine*
- *LR = Low Risk group*
- *IR = Intermediate Risk group*
- *HR = High Risk group*
- *PNP = Peripheral NeuroPathy measurement (physical examination)*
- *PK = PharmacoKinetic measurement (blood sampling)*
- *QoL = Quality of Life measurment (questionnaires)*
- *Costs = medical costs measurement (questionnaire)*
- *TE = Therapeutic Effectiveness (readily available data provided by Dutch Childhood Oncology Group)*

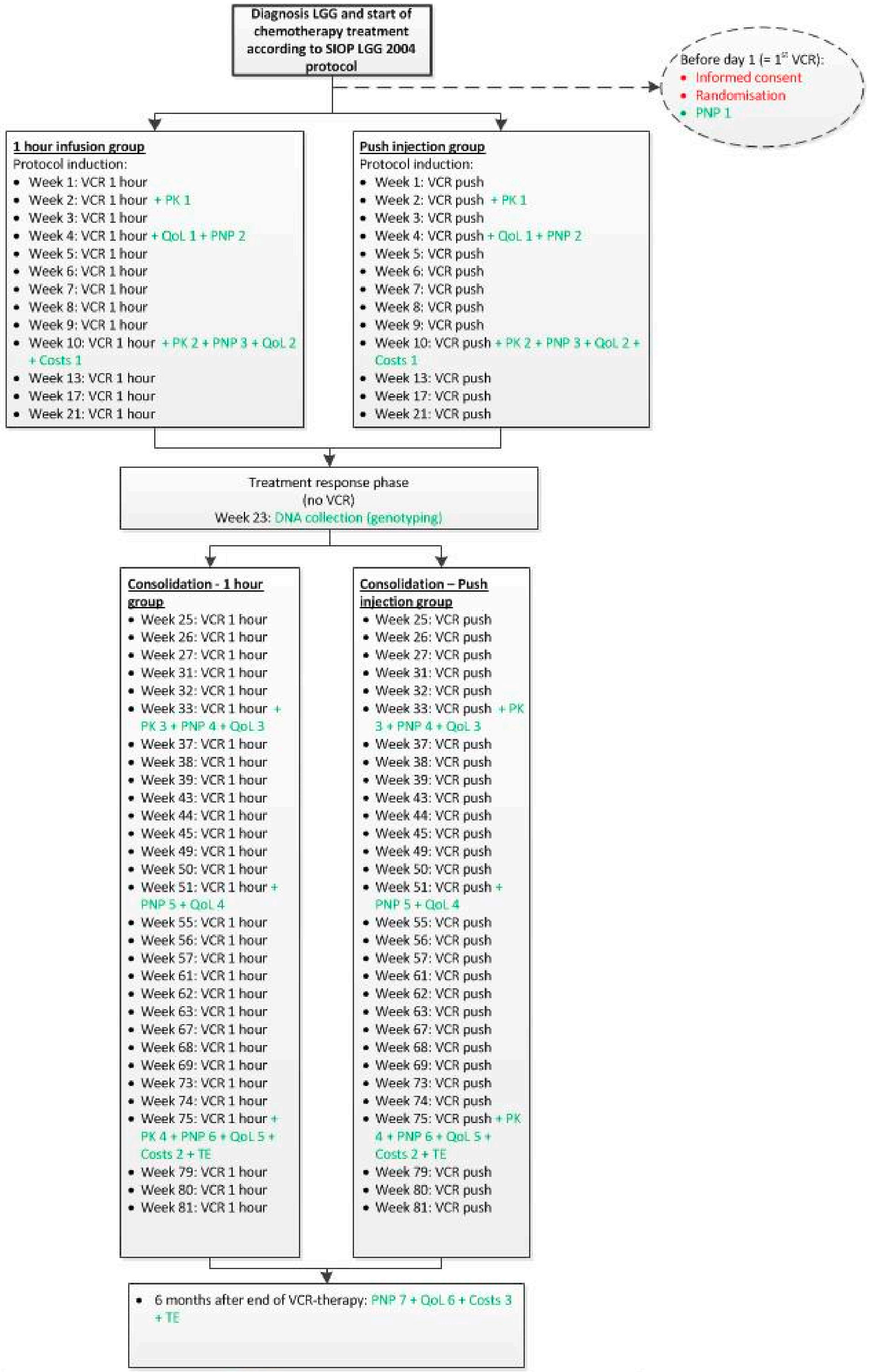

List of abbreviations:

- *VCR = Vincristine*
- *LGG = Low Grade Glioma*
- *SIOP = International Society of Paediatric Oncology*

- *PNP = Peripheral NeuroPathy measurement (physical examination)*
- *PK = PharmacoKinetic measurement (blood sampling)*
- *QoL = Quality of Life measurment (questionnaires)*
- *Costs = medical costs measurement (questionnaire)*
- *TE = Therapeutic Effectiveness (readily available data provided by Dutch Childhood Oncology Group)*

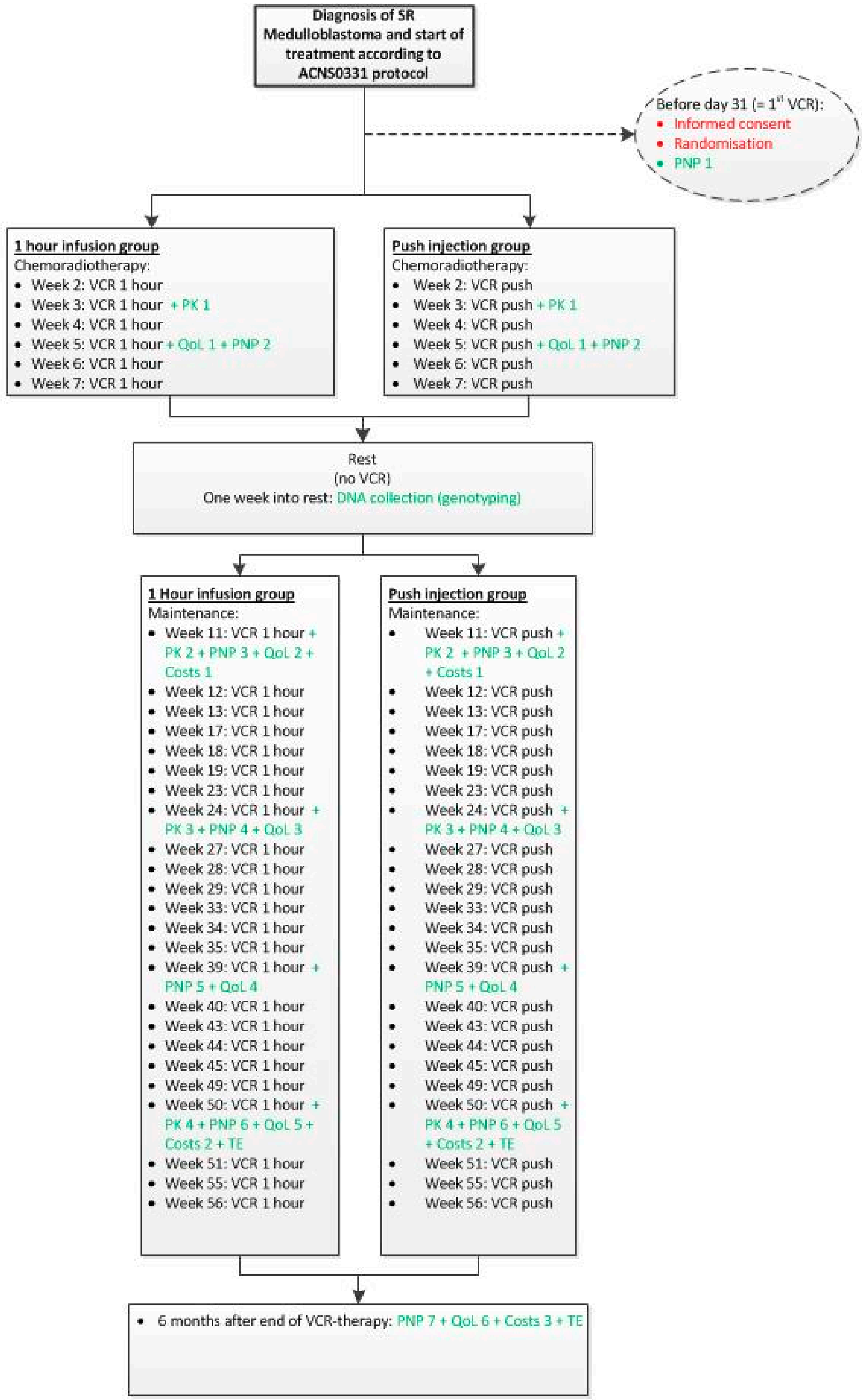

List of abbreviations:
- *VCR = Vincristine*
- *SR = Standard Risk group*

- *PNP = Peripheral NeuroPathy measurement (physical examination)*
- *PK = PharmacoKinetic measurement (blood sampling)*
- *QoL = Quality of Life measurment (questionnaires)*
- *Costs = medical costs measurement (questionnaire)*
- *TE = Therapeutic Effectiveness (readily available data provided by Dutch Childhood Oncology Group)*

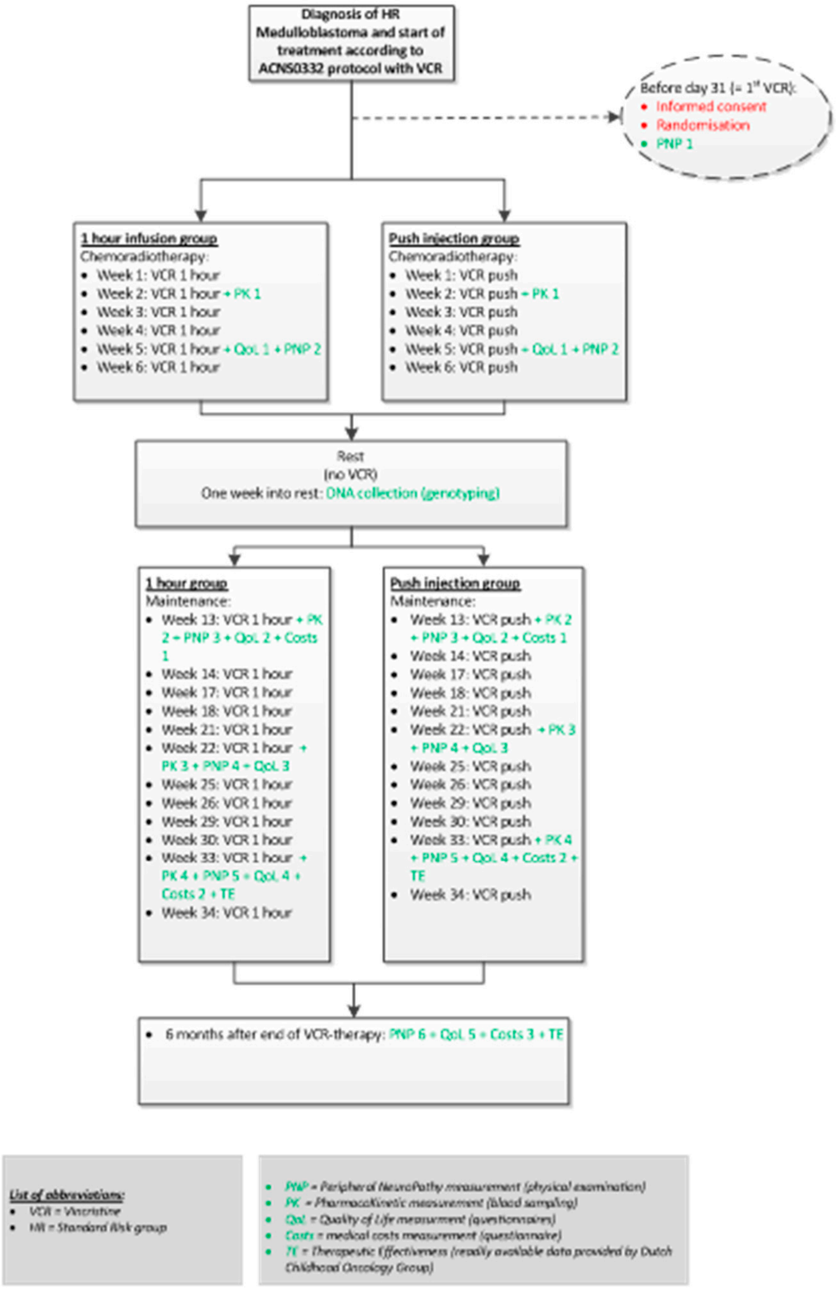
Diagnosis of HR Medulloblastoma and start of treatment according to ACNS0332 protocol with VCR
Before day 31 (= 1st VCR):
Informed consent
Randomisation
PNP 1
1 hour infusion group
Chemoradiotherapy:
Week 1: VCR 1 hour
Week 2: VCR 1 hour + PK 1
Week 3: VCR 1 hour
Week 4: VCR 1 hour
Week 5: VCR 1 hour + QoL 1 + PNP 2
Week 6: VCR 1 hour
Push injection group
Chemoradiotherapy:
Week 1: VCR push
Week 2: VCR push + PK 1
Week 3: VCR push
Week 4: VCR push
Week 5: VCR push + QoL 1 + PNP 2
Week 6: VCR push
Rest
(no VCR)
One week into rest: DNA collection (genotyping)
1 hour group
Maintenance:
Week 13: VCR 1 hour + PK 2 + PNP 3 + QoL 2 + Costs 1
Week 14: VCR 1 hour
Week 17: VCR 1 hour
Week 18: VCR 1 hour
Week 21: VCR 1 hour
Week 22: VCR 1 hour + PK 3 + PNP 4 + QoL 3
Week 25: VCR 1 hour
Week 26: VCR 1 hour
Week 29: VCR 1 hour
Week 30: VCR 1 hour
Week 33: VCR 1 hour + PK 4 + PNP 5 + QoL 4 + Costs 2 + TE
Week 34: VCR 1 hour
Push injection group
Maintenance:
Week 13: VCR push + PK 2 + PNP 3 + QoL 2 + Costs 1
Week 14: VCR push
Week 17: VCR push
Week 18: VCR push
Week 21: VCR push
Week 22: VCR push + PK 3 + PNP 4 + QoL 3
Week 25: VCR push
Week 26: VCR push
Week 29: VCR push
Week 30: VCR push
Week 33: VCR push + PK 4 + PNP 5 + QoL 4 + Costs 2 + TE
Week 34: VCR push
6 months after end of VCR-therapy: PNP 6 + QoL 5 + Costs 3 + TE
List of abbreviations:
VCR = Vincristine
HR = Standard Risk group
PNP = Peripheral NeuroPathy measurement (physical examination)
PK = PharmacoKinetic measurement (blood sampling)
QoL = Quality of Life measurement (questionnaires)
Costs = medical costs measurement (questionnaire)
TE = Therapeutic Effectiveness (readily available data provided by Dutch Childhood Oncology Group)

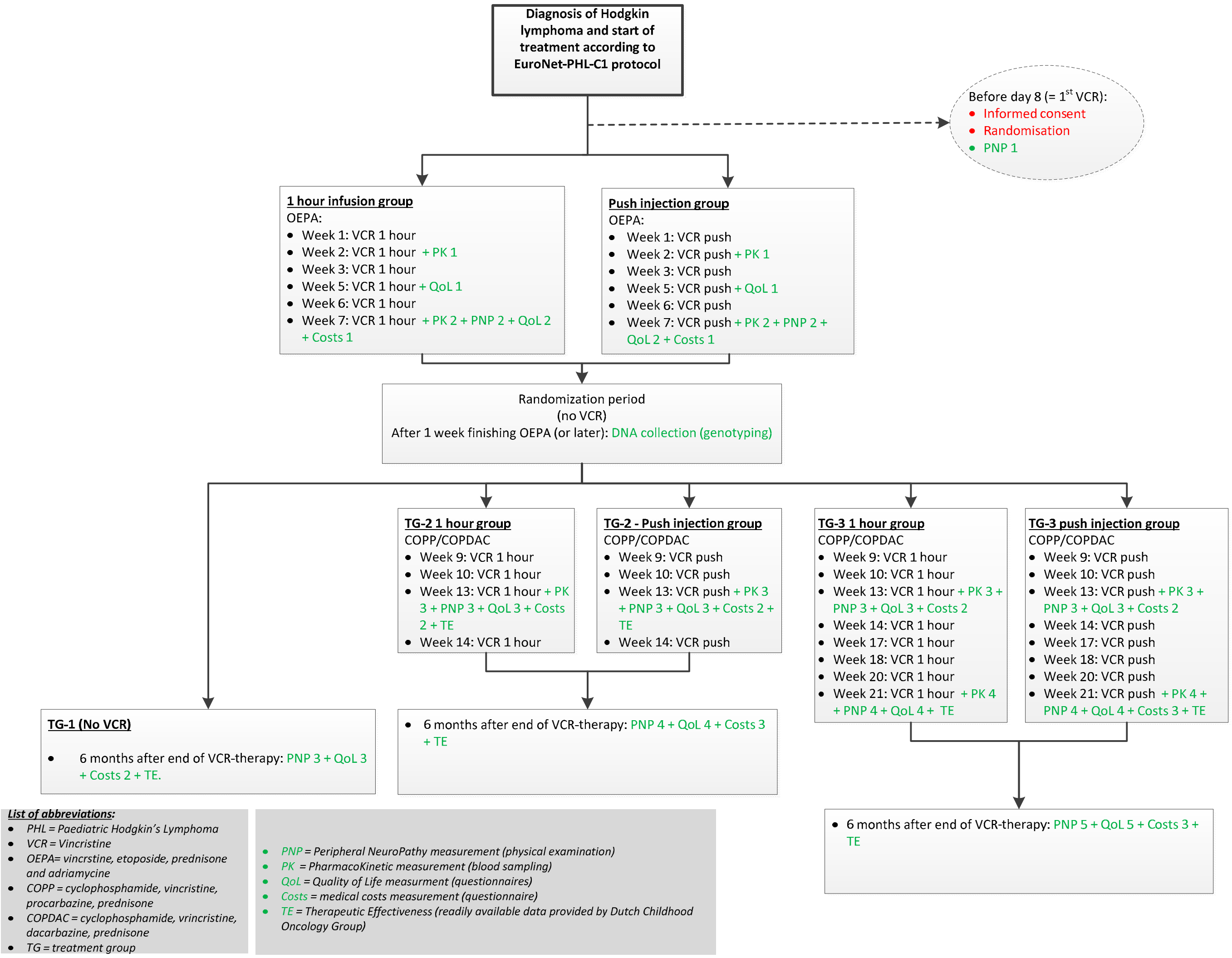

Diagnosis of Hodgkin lymphoma and start of treatment according to EuroNet-PHL-C1 protocol
Before day 8 (= 1st VCR):
Informed consent
Randomisation
PNP 1
1 hour infusion group
OEPA:
Week 1: VCR 1 hour
Week 2: VCR 1 hour + PK 1
Week 3: VCR 1 hour
Week 5: VCR 1 hour + QoL 1
Week 6: VCR 1 hour
Week 7: VCR 1 hour + PK 2 + PNP 2 + QoL 2 + Costs 1
Push injection group
OEPA:
Week 1: VCR push
Week 2: VCR push + PK 1
Week 3: VCR push
Week 5: VCR push + QoL 1
Week 6: VCR push
Week 7: VCR push + PK 2 + PNP 2 + QoL 2 + Costs 1
Randomization period
(no VCR)
After 1 week finishing OEPA (or later): DNA collection (genotyping)
TG-2 1 hour group
COPP/COPDAC
Week 9: VCR 1 hour
Week 10: VCR 1 hour
Week 13: VCR 1 hour + PK 3 + PNP 3 + QoL 3 + Costs 2 + TE
Week 14: VCR 1 hour
TG-2 - Push injection group
COPP/COPDAC
Week 9: VCR push
Week 10: VCR push
Week 13: VCR push + PK 3 + PNP 3 + QoL 3 + Costs 2 + TE
Week 14: VCR push
TG-3 1 hour group
COPP/COPDAC
Week 9: VCR 1 hour
Week 10: VCR 1 hour
Week 13: VCR 1 hour + PK 3 + PNP 3 + QoL 3 + Costs 2
Week 14: VCR 1 hour
Week 17: VCR 1 hour
Week 18: VCR 1 hour
Week 20: VCR 1 hour
Week 21: VCR 1 hour + PK 4 + PNP 4 + QoL 4 + TE
TG-3 push injection group
COPP/COPDAC
Week 9: VCR push
Week 10: VCR push
Week 13: VCR push + PK 3 + PNP 3 + QoL 3 + Costs 2
Week 14: VCR push
Week 17: VCR push
Week 18: VCR push
Week 20: VCR push
Week 21: VCR push + PK 4 + PNP 4 + QoL 4 + Costs 3 + TE
TG-1 (No VCR)
6 months after end of VCR-therapy: PNP 3 + QoL 3 + Costs 2 + TE.
6 months after end of VCR-therapy: PNP 4 + QoL 4 + Costs 3 + TE
6 months after end of VCR-therapy: PNP 5 + QoL 5 + Costs 3 + TE
List of abbreviations:
PHL = Paediatric Hodgkin's Lymphoma
VCR = Vincristine
OEPA= vincrstine, etoposide, prednisone and adriamycine
COPP = cyclophosphamide, vincristine, procarbazine, prednisone
COPDAC = cyclophosphamide, vrincristine, dacarbazine, prednisone
TG = treatment group
PNP = Peripheral NeuroPathy measurement (physical examination)
PK = PharmacoKinetic measurement (blood sampling)
QoL = Quality of Life measurment (questionnaires)
Costs = medical costs measurement (questionnaire)
TE = Therapeutic Effectiveness (readily available data provided by Dutch Childhood Oncology Group)

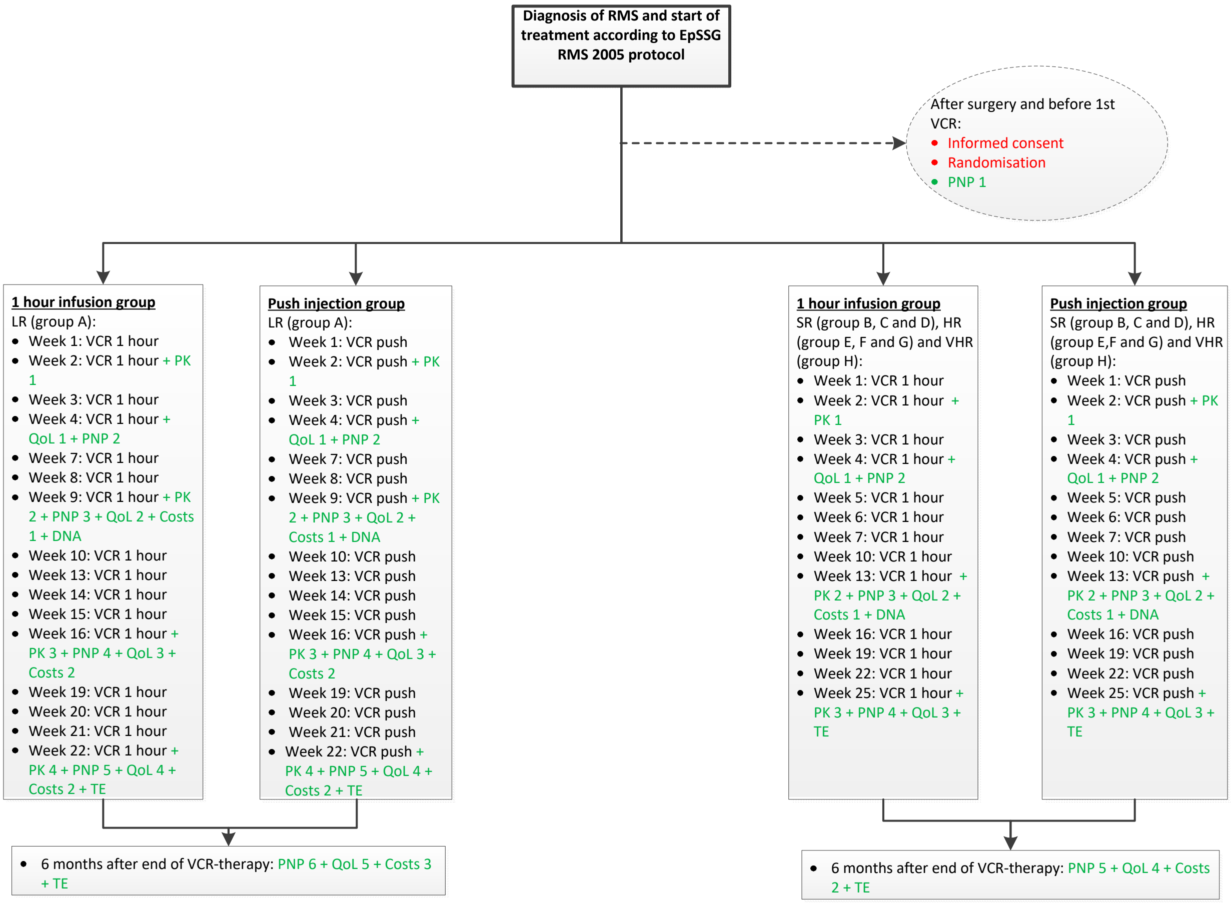

List of abbreviations:

- *RMS = rhabdomyosarcoma*
- *EPSSG = European sort tissue sarcoma study group*
- *LR = low-risk group*
- *SR = intermediate-risk group*
- *HR = high-risk group*
- *VHR = very-high risk group*
- *VCR = Vincristine*

- *PNP = Peripheral NeuroPathy measurement (physical examination)*
- *PK = PharmacoKinetic measurement (blood sampling)*
- *QoL = Quality of Life measurment (questionnaires)*
- *Costs = medical costs measurement (questionnaire)*
- *TE = Therapeutic Effectiveness (readily available data provided by Dutch Childhood Oncology Group)*

Vincristine-Induced Neuropathy in children with CAncer (the VINCA study)

RESEARCH PROTOCOL

version 5.3 –January 3, 2017

VU medisch centrum

PROTOCOL TITLE
Vincristine-induced peripheral neuropathy in paediatric oncology patients: comparing one-hour infusions with short-term infusions (the VINCA study)

**Protocol ID**	***2000790***
**Short title**	***The VINCA study (Vincristine-Induced Neuropathy in children with CAncer)***
**EudraCT number**	***2014-001561-27***
**Version**	**5.3**
**Date**	**January 3rd 2017**
**Project leader**	***Prof. dr. G.J. Kaspers<br>VU University Medical Center Amsterdam (VUmc)<br>Dep. of Paediatrics, div. of Oncology-Haematology<br>P.O. box 7057, 1007 MB, Amsterdam<br>Telephone +31 20 4442420, Fax +31 20 4442422<br>E-mail: gjl.kaspers@vumc.nl***
**Principal investigator(s) (in Dutch: hoofdonderzoeker/ uitvoerder)**	***Dr. M.H. van den Berg<br>VUmc, dep. of Paediatrics, div. of Oncology-Haematology<br>P.O. box 7057, 1007 MB, Amsterdam<br>Telephone +31 20 444 6021, Fax +31 20 444 5122<br>E-mail: mh.vandenberg@vumc.nl***
**Sponsor (in Dutch: verrichter/opdrachtgever)**	***Prof. dr. G.J. Kaspers<br>VUmc, dep. of Paediatrics, div. of Oncology-Haematology<br>P.O. box 7057, 1007 MB, Amsterdam<br>Telephone +31 20 4442420, Fax +31 20 4442422<br>E-mail: gjl.kaspers@vumc.nl***
**Subsidising party**	***ZonMw***
**Independent expert (s)**	***Prof. dr. A.A. van de Loosdrecht<br>VUmc, dep. of Hematology<br>P.O. box 7057, 1007 MB, Amsterdam<br>tel: +31 20 4441954<br>E-mail:*** a.vandeloosdrecht@vumc.nl
**Laboratory sites**	***Dr. J. Cloos<br>Hematology Laboratories, Cancer Center Amsterdam, VUmc<br>Tel: +31 20-4447413<br>E-mail: j.cloos@vumc.nl***
**Pharmacy**	***A.J. Wilhelm, hospital pharmacist, clinical pharmacologist<br>VUmc<br>Tel: +31 20-4443076<br>E-mail: aj.wilhelm@VUmc.nl***

PROTOCOL SIGNATURE SHEET

**Name**	**Signature**	**Date**
**Head of Department:**	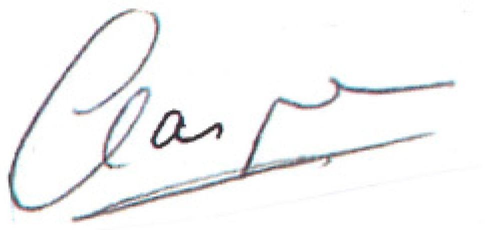<br>**Prof. dr. G.J. Kaspers**<br>**Head of Pediatric Oncology/Hematology, VU University Medical Center**	**03-01-2017**
**Principal Investigator:**	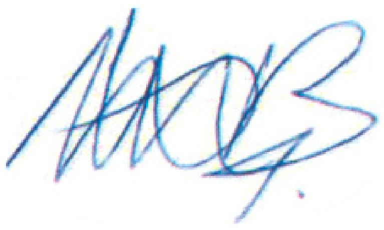<br>**Dr. M.H. van den Berg**<br>**Post-doctoral researcher Pediatric Oncology/ Hematology , VU University Medical Center**	**03-01-2017**

TABLE OF CONTENTS

1. INTRODUCTION AND RATIONALE 18
2. OBJECTIVES 20
3. STUDY DESIGN 21
4. STUDY POPULATION 22
4.1 Population (base) 22
4.2 Inclusion criteria 22
4.3 Exclusion criteria 22
4.4 Sample size calculation 22
5. TREATMENT OF SUBJECTS 24
5.1 Investigational treatment 24
5.1.1 Treatment of ALL 24

5.2 Use of co-intervention 27
5.3 Escape medication 28
6. INVESTIGATIONAL PRODUCT 29
6.1 Name and description of investigational product(s) 29
6.2 Summary of findings from non-clinical studies 29
6.3 Summary of findings from clinical studies 29
6.4 Summary of known and potential risks and benefits 29
6.5 Description and justification of route of administration and dosage 29
6.6 Dosages, dosage modifications and method of administration 29
6.7 Preparation and labelling of Investigational Medicinal Product 30
7. NON-INVESTIGATIONAL PRODUCT 31
8. METHODS 32
8.1 Study outcomes 32
8.1.1 Main study outcome 32
8.1.2 Secondary study outcomes 32
8.2 Randomization and treatment allocation 36
8.3 Study procedures 36
8.4 Withdrawal of individual subjects 38
8.5 Replacement of individual subjects after withdrawal 39
8.6 Follow-up of subjects withdrawn from treatment 39
8.7 Premature termination of the study 39
9. SAFETY REPORTING 40
9.1 Section 10 WMO event 40
9.2 AEs, SAEs and SUSARs 40
9.2.1 Adverse events (AEs) 40
9.2.2 These mentioned AEs are marked as AEs of special interest. Serious adverse events (SAEs) 40
9.2.3 Suspected unexpected serious adverse reactions (SUSARs) 41
9.3 Annual safety report 42
9.4 Follow-up of adverse events 42
9.5 Safety Committee 42
10. STATISTICAL ANALYSIS 43
10.1 Analyses of primary and secondary outcomes 43
10.2 Interim analysis 44
11. ETHICAL CONSIDERATIONS 45
11.1 Regulation statement 45
11.2 Recruitment and consent 45
11.3 Objection by minors or incapacitated subjects (if applicable) 45
11.4 Benefits and risks assessment, group relatedness 45
11.5 Compensation for injury 46
11.6 Incentives 46
12. ADMINISTRATIVE ASPECTS, MONITORING AND PUBLICATION 47
12.1 Handling and storage of data and documents 47
12.2 Monitoring and Quality Assurance 47

12.3 Amendments 47
12.4 Annual progress report 47
12.5 End of study report 47
12.6 Public disclosure and publication policy 47
13. STRUCTURED RISK ANALYSIS 48
14. REFERENCES 48
15. List of attachments 52
15.1 VCR preparation protocol of VUmc pharmacy (one hour) (in Dutch) 52
15.2 VCR preparation protocol of VUmc pharmacy (bolus injection/infusion) (in Dutch) 53
15.3 Example of a label (in Dutch) 54
15.4 Study measurement schedule within ALL-11 and EsPhALL protocol 55
15.5 Study measurement schedule within SIOP 2001 protocol 56
15.6 Study measurement schedule within ACNS0331 protocol 58
15.7 Study measurement schedule within ACNS0332 protocol 59
15.8 Study measurement schedule within SIOP LGG 2004 protocol 60
15.9 Study measurement schedule within EuroNet-PHL-C1 or C2* protocol 61
15.10 Study measurement schedule within EpSSG RMS 2005 protocol 62

LIST OF ABBREVIATIONS AND RELEVANT DEFINITIONS

**ABR**	**ABR form, General Assessment and Registration form, is the application form that is required for submission to the accredited Ethics Committee (In Dutch, ABR = Algemene Beoordeling en Registratie)**
**AE**	**Adverse Event**
**ALL**	**Acute Lymphoblastic Leukemia**
**AR**	**Adverse Reaction**
**CA**	**Competent Authority**
**CCMO**	**Central Committee on Research Involving Human Subjects; in Dutch: Centrale Commissie Mensgebonden Onderzoek**
**CV**	**Curriculum Vitae**
**DCOG**	**Dutch Childhood Oncology Group; in Dutch: SKION**
**DSMB**	**Data Safety Monitoring Board**
**EFS**	**Event Free Survival**
**EudraCT**	**European drug regulatory affairs Clinical Trials**
**GCP**	**Good Clinical Practice**
**HL**	**Hodgkin Lymphoma**
**HRMB**	**High Risk Medulloblastoma**
**IB**	**Investigator’s Brochure**
**IC**	**Informed Consent**
**IMP**	**Investigational Medicinal Product**
**IMPD**	**Investigational Medicinal Product Dossier**
**LGG**	**Low-Grade Glioma**
**METC**	**Medical research ethics committee (MREC); in Dutch: medisch ethische toetsing commissie (METC)**
**NB**	**Nephroblastoma**
**PK**	**Pharmacokinetics**
**PNP**	**Peripheral NeuroPathy**
**RMS**	**Rhabdomyosarcoma**
**QoL**	**Quality of Life**
**(S)AE**	**(Serious) Adverse Event**
**SRMB**	**Standard Risk Medulloblastoma**
**SPC**	**Summary of Product Characteristics (in Dutch: officiële productinfomatie IB1-tekst)**
**Sponsor**	**The sponsor is the party that commissions the organisation or performance of the research, for example a pharmaceutical company, academic hospital, scientific organisation or investigator. A party that provides funding for a study but does not commission it is not regarded as the sponsor, but referred to as a subsidising party.**
**SUSAR**	**Suspected Unexpected Serious Adverse Reaction**
**VCR**	**Vincristine**
**Wbp**	**Personal Data Protection Act (in Dutch: Wet Bescherming Persoonsgevens)**
**WMO**	**Medical Research Involving Human Subjects Act (in Dutch: Wet Medisch-wetenschappelijk Onderzoek met Mensen**

SUMMARY

Rationale: Vincristine (VCR) is a commonly used chemotherapeutic drug in the treatment of childhood cancer. The main dose-limiting side effect of VCR is peripheral neuropathy (PNP). PNP is often seen in the form of weakness of lower limbs, areflexia, neuropathic pain, and/or sensory loss. The quality of life of children who suffer from VCR-induced PNP is severely affected.

There is a lack of information regarding the optimal therapeutic dosing and method of administration of VCR for children with cancer. High peak plasma concentrations seem to be correlated with PNP. However, the exact mechanism underlying VCR-induced PNP is not clear.

The study is set up as a prospective, multi-center, open-label, randomized controlled trial with a duration of 42 months. In the Netherlands almost all children diagnosed with cancer are treated according to standardized treatment protocols, with many of these protocols consisting of multiple administrations of VCR. Patients who participate in the current study trial will receive all VCR administrations of their treatment protocol either by short-term infusions (1-5 minutes) or by one-hour infusions. Study measurements will be performed at several points in time.

Objective: This study aims to investigate whether the administration of VCR in children with cancer by one-hour infusions, resulting in lower peak plasma concentrations, leads to less PNP compared to short-term infusions. In addition, quality of life, (non-)medical costs, and treatment efficacy associated with both administration methods will be evaluated. Moreover, it will be investigated whether other factors, such as pharmacokinetics and genetic susceptibility to drug-induced side-effects, also influence the degree of PNP.

Study design: The study is set up as a prospective randomized controlled trial.

Study population: The study population consists of children aged 2-18 years who are about to start VCR therapy for a newly diagnosed oncologic disease. Patients who will receive at least 6 VCR administrations during their treatment period are deemed eligible for the study.

Intervention: Patients will be randomized into two groups. Patients in group A (the short term administration group) will receive all VCR administrations during their treatment period by 1-5 minute infusions. Patients in group B (the long term administration group) will receive all VCR administrations by one-hour infusions.

Main study endpoints: The primary outcome of the study is PNP. Secondary endpoints include quality of life, medical costs, and treatment efficacy. In addition, we will study pharmacokinetic parameters and genetic polymorphisms in relation to PNP.

Nature and extent of the burden and risks associated with participation, benefit and group relatedness: The burden for participating patients and their guardians is considered to be low. The risk-benefit analysis for the study shows a favourable risk profile. The investigational medicinal product (IMP) that is studied is VCR, a drug which is widely used. Moreover, the investigated methods of administration (bolus injection and 1 hour infusion) are both being applied in children in clinical practice. Study measurements will be performed at several points in time. At these time points patients are asked to do the following: 1. undergo a physical neurological examination (3-7 measurements in total, 20 minutes on average per measurement); 2. fill out questionnaires (QoL: 3-7 measurements in total, 15-20 minutes on average per measurement; costs: 1-3 measurements in total; 10 minutes on average per measurement); 3. provide blood samples (1-4 pharmacokinetic measurements in total, 8 samples per measurement). Clinical measurements and blood sampling will be performed on days the patient has to visit the clinic for treatment purposes; questionnaires will be given to the patients/guardians and can be filled out at home. Although it is not the primary aim of this study, participants can benefit from the effects of the intervention. The benefit could be less PNP, resulting in improved quality of life, also for all future childhood cancer patients treated with VCR.

INTRODUCTION AND RATIONALE

Cancer is the most common cause of death in childhood with around 650 newly diagnosed children each year. Treatment of some of these types of cancer consist of multiple administrations of the chemotherapeutic drug vincristine (VCR)(1). A major adverse effect of VCR exposure is neurotoxicity, characterized by autonomic and peripheral sensory-motoric neuropathy and occurring in at least 30% to 40% of treated patients (2-4). PNP is often seen in the form of weakness of lower limbs, areflexia, neuropathic pain, and/or sensory loss. Autonomic neuropathy presents as constipation, urinary retention, and/or encephalopathy. It has been demonstrated that chemotherapy-induced PNP among children with cancer deteriorates the quality of life (QoL) of these patients, mainly caused by a decreased motor function (foot drop, walking difficulties, and balance and gait disorders) as well as the presence of neuropathic pain (4-6). Moreover, lifelong sequelae of PNP are common in survivors of childhood cancer (7-10).

To date, no specific preventive or curative treatment is available to manage VCR-induced PNP. In some cases gabapentin or a similar drug is given in order to diminish neuropathic pain, but most often the only way to decrease PNP is to reduce VCR dosages, increase intervals, or discontinue VCR treatment, thereby potentially impairing treatment efficacy. As the survival rate of pediatric cancer patients has greatly increased in the past decades, QoL measures become critical secondary goals in the fight against cancer. The neuropathies induced by successful treatment mandate investigations into strategies to reduce adverse effects while maintaining treatment efficacy.

Although the mechanism of VCR-induced PNP is not fully understood, it appears to be a dose-dependent phenomenon with high peak plasma VCR concentrations correlating with PNP (11, 12). However, despite decades of widespread clinical use, there is a lack of information regarding the optimal therapeutic dosing and method of administration of VCR (13, 14). The SPC states that VCR can be administered both by means of infusion or a bolus (push) injection of at least 1 minute. In clinical practice both short-term infusions and infusions lasting 15-60 minutes are being used in pediatric oncology patients. For the current study short-term infusions are defined as any administration (infusions or injections) lasting minimally 1 and maximally 5 minutes. Since applying one-hour infusions instead of short-term infusions or 15-30 minutes infusions prevents the occurrence of high peak concentrations, we hypothesize that one-hour infusions of VCR will lead to less PNP than short-term infusions in children with cancer. If this indeed appears to be the case, the use of one-hour VCR infusions will reduce both the number of pediatric cancer patients suffering from VCR-induced PNP as well as the severity of PNP of those affected. In the past a few studies have investigated the neurotoxic side effects of continuous infusion. However, these studies applied very long infusions only (4-5 days), and all infusions were preceded by a bolus injection (14-18). Moreover, studies among pediatric oncology populations are scarce. As a consequence, information on the occurrence of PNP associated with one-hour infusions in pediatric patients is lacking.

In order to properly investigate the relationship between PNP outcomes in children receiving VCR by means of the two administration methods, it is crucial for the proposed randomized study to also take into account the pharmacokinetic profile of VCR in these patients. It is known that pharmacokinetic characteristics of children receiving VCR vary largely from patient to patient (19, 20). In this study several pharmacokinetic parameters, such as volume of distribution, intercompartmental clearance, and total body clearance will be assessed. In this way, VCR pharmacokinetics of both administration methods can be compared and related to PNP outcomes. However, in order to appropriately evaluate and interpret VCR pharmacokinetic measures in the current study, one should also relate them to the genetic profiles of the study participants. VCR is metabolized by the cytochrome P450 system in the liver. It is known that the CYP3A family of enzymes plays an important role in the metabolism of VCR (21). More specifically, metabolic clearance appears to be significantly greater with CYP3A5 than with CYP3A4 polymorphisms (22). However, VCR pharmacokinetics are

influenced by other genetic factors as well, such as variations in the MDR-1 (or ABCB1) and the MAPT gene (21, 23). However, their relation with neurotoxicity remains to be established (24-28). This study will help to further identify whether the afore-mentioned genetic factors indeed are associated with VCR-induced PNP.
Furthermore, previous studies have identified genetic mutations that influence the patient's susceptibility to VCR-induced PNP by other ways than through affecting the pharmacokinetics of VCR (29-31). For example, it has been demonstrated that genes involved in cell cycle and proliferation are related to VCR-induced PNP (30). Although the number of studies investigating the genetic factors that are associated with the development of VCR-induced PNP is limited, a list of candidate genes and single nucleotide polymorphisms (SNPs) is available (see chapter 8.1.2).

In conclusion, it can be stated that the results of this study will contribute to an effective use of VCR with minimal PNP in children treated for cancer. In case it is demonstrated that the degree of PNP in children with cancer is reduced by applying one-hour VCR infusions instead of short-term infusions, the QoL of these children will most likely improve. In addition, by relating PNP outcomes to several genetic factors known to be associated with the development of VCR-induced PNP, it will be possible to further investigate why children respond differently to both administration methods. Moreover, in case one-hour infusions of VCR appears to cause less PNP, this will not only positively influence QoL, it will also likely lead to a reduction of medical costs. Calhoun et al. demonstrated that chemotherapy-induced neurotoxicity of adult cancer patients indeed is associated with substantial direct and indirect medical costs (32). Although comparable studies in children have not been conducted so far, costs savings may very well be accomplished. Less PNP in children with cancer will for example lead to less prescriptions of medication to decrease neuropathic pain. Furthermore, it is known that for example 30% of children with ALL are referred to a physiotherapist, mainly because of adverse effects attributed to VCR (33). A reduction in PNP will lead to less children being referred to physiotherapeutic treatments and less prescriptions of medical devices such as wheel chairs, braces or splints; all of which will lead to a decrease in direct medical costs. Finally, results of this study will be relevant for future children receiving VCR as part of their anti-cancer treatment.

OBJECTIVES

Primary Objective:

- To evaluate whether the administration of vincristine (VCR) in children with cancer by one-hour infusions leads to less peripheral neuropathy (PNP) compared to the administration by short-term infusions lasting 1-5 minutes.

Secondary objectives:

- To evaluate whether the administration of VCR in children with cancer by one-hour infusions leads to better self-reported quality of life (QoL) compared to the administration by short-term infusions lasting 1-5 minutes.

- To evaluate whether the administration of VCR in children with cancer by one-hour infusions leads to less direct and indirect medical costs compared to the administration by short-term infusions lasting 1-5 minutes.

- To evaluate whether the administration of VCR in children with cancer by one-hour infusions leads to equal or better treatment efficacy compared to the administration by short-term infusions lasting 1-5 minutes.

- To compare the pharmacokinetic parameters of patients receiving both administration methods and to relate these parameters to the degree of PNP.

- To validate or confirm the relationship between SNPs known to be associated with VCR-induced PNP (either through influencing pharmacokinetics of VCR or through genetically increased susceptibility) and the degree of PNP of patients receiving both administration methods.

3. STUDY DESIGN

The study is set up as a prospective, multi-center, open-label, randomized controlled trial. In the Netherlands almost all children diagnosed with cancer are treated according to standardized treatment protocols. In the current study participants will be randomly allocated to either receive all VCR administrations of these protocols by short-term infusions lasting 1-5 minutes or by one-hour infusions. Study measurements will be performed at several points in time (depending of duration of VCR treatment): at baseline, one to five times during the VCR-therapy or at the end of therapy, and 6 months after the end of VCR-therapy (see also chapter 6). The main setting of the study will be the VU University Medical Center Amsterdam, department of pediatric oncology/ hematology.
The SPC of VCR states that VCR can be administered by means of infusion or a bolus (push) injection, the latter in at least one minute. Moreover, in clinical practice both short-term infusions and 1 hour infusions are used in pediatric oncology patients.

The overall time frame of the study will encompass 42 months (3.5 years):

- Preparation phase: 4 months (acquiring ethical approval, discussing study protocol with participation centers, training assessors, developing patient information brochures)
- Execution phase: 32 months (performing measurements)
- Data analyses and reporting: 6 months

4. STUDY POPULATION

Population (base)

The study population will consist of children who are about to start VCR therapy for a newly diagnosed cancer and whose treatment protocol consists of at least 6 VCR administrations of which 4 are administered in a time period of 6 weeks maximum (34).These children include those children treated for:

- ALL according to the ALL-11 protocol (34) (or its successor in case the VCR schedule is similar) or to the EsPhALL (35) protocol (in case of Philadelphia chromosome positive ALL) (or its successor in case the VCR schedule is similar);
- nephroblastoma (NB) treated according to the SIOP Wilms 2001 protocol (36) (or its successor in case the VCR schedule is similar);
- standard risk medulloblastoma (SRMB) treated according to the ACNS0331 protocol (37) (or its successor in case the VCR schedule is similar);
- high risk medulloblastoma (HRMB) treated according to the ACNS0332 protocol (38) (or its successor in case the VCR schedule is similar);
- low-grade glioma (LGG) treated according to the SIOP LGG 2004 protocol (39) (or its successor in case the VCR schedule is similar);
- Hodgkin lymphoma (HL) treated according the EuroNet-PHL-C1 protocol (40) (or its successor in case the VCR schedule is similar);
- rhabdomyosarcoma (RMS) treated according to the EpSSG RMS 2005 protocol (41) (or its successor in case the VCR schedule is similar).

Inclusion criteria

- age between 2 and 18 years.
- treated for cancer according to a treatment protocol which includes at least 6 administrations of VCR of which 4 are administered within a maximum period of 6 weeks;
- diagnosed with a cancer diagnosis of which the incidence in the Netherlands is more than 5 children per year;
- written informed consent.

Exclusion criteria

- patient or parent refusal;
- history of PNP or other pre-existing or disease-related sensory or motor neurologic conditions;
- pre-existing severe mental retardation;
- having parents/ guardians who are unable to communicate in the Dutch language.

Sample size calculation

The calculation of the required sample size for the study is based on the primary outcome measure, i.e. PNP. In a previous study which evaluated the degree of neurotoxicity in a large group of pediatric ALL-patients by applying the same scoring method as described in our study (the NCI CTCAE method) the average maximum grade of neurotoxicity was (2.43 ± 1.07) (21, 42). Since, besides ALL patients, patients with other cancer diagnosis will be included as well, we expect the standard deviation to be slightly larger than 1.07, namely 1.3. For our study it was decided to consider a difference in PNP score between the two intervention groups of at least 1.0 as a clinically relevant difference. Using these data it was calculated that 35 patients in each intervention group are needed (alpha=5% and power=90%). To compensate for a drop-out rate of 25% and occasional missing samples, it was decided to include 44 patients in each group (88 patients in total).

As stated above, the study population consists of patients treated for ALL, NB, SRMB, HRMB, LGG, HL and RMS. Approximately 120 children a year are diagnosed with ALL in the Netherlands, whereas 17 children are diagnosed with NB, 23 with SRMB and HRMB, 53 with LGG, 30 with HL and 14 with RMS(1). Patient inclusion for the study will start in VU University Medical Center Amsterdam. However, in order to meet the defined time schedule and patient numbers, participation of other centers is imperative.

Moreover, five Dutch pediatric oncology centers will participate in the study. As soon as patient inclusion for other ongoing studies has been closed, inclusion for the current study will be possible in the following centers:

- Erasmus Medical Center - Sophia Children’s Hospital, Rotterdam, Netherlands,
- Emma Children’s Hospital/Academic Medical Center, Amsterdam, Netherlands,
- Beatrix Children’s Hospital/ University Medical Center, Groningen, Netherlands,
- Radboud University Nijmegen Medical Center, Nijmegen, Netherlands
- Princess Maximá Center for Pediatric Oncology, Utrecht, Netherlands

Ethical approval for participation of the Dutch centers will be amended somewhere at the end of 2015 or in the beginning of 2016 at the METC of VUmc Amsterdam.

TREATMENT OF SUBJECTS

Investigational treatment

This study investigates whether the degree of PNP differs between different administration methods of VCR given during treatment for pediatric ALL, NB, SRMB, HRMB, LGG, HL and RMS. The VCR dosage as prescribed in the treatment protocols of these types of cancer varies between 1.5 mg/m^2 and 2.0 mg/m^2 (depending on the diagnosis and age of the patient) with a maximum of 2.0 mg/m^2 per administration. In the current study no changes will be made regarding the dose, the number or the timing of the VCR administrations as prescribed by the concerning protocol, unless the medical situation of the participants requires to do so (see also chapter 5.2 and 5.3). The only ‘deviation’ from the various protocols that will be made, is the fact that half of the participating patients will receive one hour infusions of VCR and the other half will receive their VCR by means of short-term infusions, as determined by randomization. The SPC states that “VCR can be administered intravenously either by means of infusion or bolus injection of at least 1 minute through the line of a flowing intravenous cannula". As a consequence, in clinical practice VCR is administered both through short-term infusions as well as prolonged infusions (ranging from 15 to 60 minutes) in pediatric oncology patients. Which method is being used depends merely on local center’s logistical/practical considerations.

In the next paragraphs a summary is given of the VCR administrations as well as the most important other chemotherapeutic agents within the different treatment protocols

5.1.1 Treatment of ALL

5.1.1.1. Treatment of ALL according to ALL-11 protocol
Treatment for pediatric ALL according to the ALL-11 protocol involves an induction phase (protocol IA and IB) that starts with administration of prednisone in the first week, followed by 4 VCR and daunorubicin administrations (once a week). At different timepoints pegylated aspariganase, cyclophosphamide, cytarabine, mercaptopurine and intrathecal medication is given. This phase is followed by CNS-directed therapy (protocol M) without VCR therapy at which several intrathecal medication and high dose methotrexate is given. Also daily mercaptopurine is given. Subsequently, based on the risk group in which the patient is characterized, treatment continues as follows:

- standard risk group (SR): protocol IV that consists of 2 VCR administrations, once a week and oral dexamethasone and an individualized dosage pegylated asparaginase, followed by maintenance therapy (no VCR) at which oral methotrexate and mercaptopurine is given;
- medium risk group (MR): intensification/maintenance therapy (28 VCR administrations, once every 3 weeks). Furthermore maintenance therapy consists of different time periods at which dexamethasone, methotrexate, pegylated asparaginase, mercaptopurine and intrathecal medication is given;
- high risk group (HR): 6 HR courses of which 4 courses include 2 VCR administrations per course, followed by protocol IIA and IIB which consists 4 VCR administrations, once a week, followed by maintenance therapy (no VCR). During these HR courses, the same chemotherapeutic agents as given during maintenance therapy of the MR group patients are administered. Furthermore, this is combined with administrations of etoposide, mitoxantrone, fludarabine, idarubicin and high dose cytarabin.

The duration of the total treatment period is two years for the majority of patients. For Ikaros (IKZF1) deleted patients in the medium risk group the treatment period is extended by one year, consisting of MTX and 6-MP only. In conclusion, the amount of VCR administered

between the 3 treatment groups differs: 6 in the standard risk group, 32 in the medium risk group and 12 in the high risk group. All administrations have a 1,5 mg/m^2 dosage, except for the maintenance administrations in the medium risk group. These have a 2 mg/m^2 dosage.

5.1.1.2. Treatment of ALL according to EsPhALL protocol
Treatment of Philadelphia chromosome positive ALL is very similar to the treatment of high-risk ALL treated according to ALL-11 protocol with the addition of treatment with imatinib. Study measurements will be similar to ALL high-risk study measurements.

5.1.2 Treatment of NB
Treatment of NB according to the SIOP-2001 protocol starts with a preoperative treatment with chemotherapy of four weeks (VCR and actinomycin D). Patients with metastasis at diagnosis will receive 6 weeks pre-operative treatment with doxorubicin as additional drugs. After this preoperative phase, surgery will be performed to remove the tumor. In the postoperative stage treatment is given in all patients according to local stage and histology (for details see protocol (36)). Patients with local stage II and III and intermediate risk tumors after surgery and no metastasis will receive postoperative treatment. This consists of actinomycin D and VCR and in some cases doxorubicin.

- Stage 1 low risk will not receive further treatment and will therefore only be included for 1 follow-up measurement six months after treatment completion.
- Stage 1 intermediate risk patients will receive one cycle of actinomycin D and 4 times VCR (AV-1 regimen).
- Stage II and III low risk patients will receive 3 cycles of actinomycin D starting at week 2 after start of postoperative treatment in 3 week intervals, 8 weekly administrations of VCR and 2 administrations of doxorubicin (week 2 and 8). After 8 weeks there is a treatment pause of 2 weeks. In week 11 actinomycin D is restarted in 3 weekly intervals until week 26. VCR is also started on week 11 with 2 weekly administrations followed by a pause of a week until week 27 (AV-2 regimen).
- Stage I high risk and stage II/III intermediate risk will receive the AV-2 regimen with additional doxorubicin started on week 14 once every 6 weeks until week 26 (AVD regimen).

Stage II and III high-risk patients will receive high risk treatment that includes no VCR and will therefore be included for follow-up measurements. In conclusion, patients with stage I low risk and patients with stage II and III high-risk will receive 4 or 6 pre-operative VCR administrations, stage I intermediate will receive 8 VCR administrations and stage II and III low risk, stage I HR and stage II/II intermediate risk will receive 24 VCR administrations. All administrations have a 1,5 mg/m^2 dosage.

5.1.3 Treatment of SRMB
Treatment of SRMB according to the ACNS0331 protocol starts with surgery. Within 31 days of definitive surgery a chemo-radiotherapy phase starts. In this phase radiotherapy is combined with 6 VCR administrations weekly on week 2 until week 7. This is followed by 4 weeks of rest. After this, the maintenance phase consists of 2 different cycles: A and B. This consist of 2 cycles A, then one cycle B, then 2 cycles A, 1 cycle B, 2 cycles A and 1 cycle B.

- Duration of 1 cycle A is 6 weeks. It starts with lomustine, VCR and cisplatin on day 1 and VCR additionally on day 8 and 15, followed by a rest-phase until week 7.
- Duration of 1 cycle B is 4 weeks and starts with cyclophosphamide day 1 and 2, MESNA day 1 and 2 and VCR on day 1 and 8, followed by a rest-phase until week 5.

Total duration of maintenance with these 9 cycles will be 56 weeks. In conclusion 30 VCR administrations will be given. All administrations have a 1,5 mg/m^2 dosage.

5.1.4 Treatment of HRMB

Treatment of HRMB according to the ACNS0332 protocol starts with surgery. Chemo-radiotherapy phase must start within 31 days after diagnostic surgery. Chemo-radiotherapy phase consists of 6 weeks of chemo-radiotherapy and 6 weeks rest period.

- On day 1 of week 1 through 6 VCR is administered. Filgrastim should be administered during radiation therapy as needed.
- Maintenance therapy consists of 6 cycles in total. The duration of one cycle is four weeks. Day 1 starts with cisplatin and VCR treatment, on days 2 and 3 cyclophosphamide is administered. On day 8 VCR is administered. Filgrastim is started on day 4 and continued for at least 10 days.

In conclusion VCR is administered 18 times. All administrations have a 1,5 mg/m^2 dosage.

5.1.5 Treatment of LGG

Treatment of LGG according to the SIOP-LGG-2004 starts with a ten week induction period. This period consists of weekly VCR administrations. Furthermore on week 1, 4, 7 and 10 cisplatin is administered. This is followed by a rest-phase of 2 weeks. On weeks 13, 17 and 21 VCR and cisplatin are administered. Then a treatment response phase is following.

Consolidation therapy starts from week 25 onwards. It consists of 12 cycles of 6 weeks. Every cycle consists of 3 weekly administrations of VCR and 1 administration of carboplatin on day 1. This is followed by a rest-phase of 3 weeks until the start of a next cycle. In conclusion, VCR is administered 43 times. All administrations have a 1,5 mg/m^2 dosage.

5.1.6 Treatment of HL

Treatment of HL according to the EuroNet-PHL-C1 or C2 protocol is divided between 3 treatment groups (TG). TG-1 consists of patients with stage IA/B and stage IIA. TG-2 are patients staged I_EA/B, II_EA, II B or III A. TG-3 patients are stages II_EB, III_EA/B, III B or IV A/B

- TG-1 starts with two courses of prednisone, VCR, doxorubicin and etoposide (OEPA). Prednisone is administered daily during 15 days, Vincristine is administered on days 1, 8 and 15. Doxorubicin is administered on day 1 and 15 and etoposide on day 1 through 5. Days 16 until 28 are a treatment free interval. The next cycle starts on day 29. Patients with inadequate response will receive involved field radiotherapy. Patients in TG-1 with adequate response receive no further therapy (C1) or one cycle of COPDAC (see below).
- Patients in TG-2 also start with 2 OEPA courses after which response is evaluated. This is followed by 2 courses of prednisone, dacarbazine, VCR and cyclophosphamide (COPDAC). This course consists of prednisone on day 15 through 15, dacarbazine day 1 through 3, VCR day 1 and 8 and cyclophosphamide day 1 and 8. Days 16 until 28 are a treatment free interval. The next cycle starts on day 29. Patients with inadequate response will receive involve field radiotherapy after the end of chemotherapy. Patients in TG-3 will follow the same schedule as patients in TG-2, except they will receive 4 COPDAC courses instead of 2 COPDAC courses. They will also receive involve field radiotherapy after the end of chemotherapy in case of inadequate response.
- Patients within the C2 protocol are randomized to receive DECOPDAC instead of COPDAC. A DECOPDAC course is similar to the COPDAC course, with the addition of etoposide on day 1-3 and doxorubicin on day 1. Furthermore, prednisone is only used for 8 days. Between day 8 and 21 no treatment is administered. The following cycle starts on day 22. VCR administrations are similar between COPDAC and DECOPDAC courses are similar.

In conclusion is VCR administered 6 times in TG-1, 10 times in TG-2 and 14 times in TG-3. All administrations have a 1,5 mg/m^2 dosage.

5.1.7 Treatment of RMS

Treatment of RMS is according to the EpSSG-RMS-2005 protocol. All patients undergo initial surgery. After this, treatment group is assessed.

- Low risk (LR) (subgroup A) patients will be treated with courses of VCR and actinomycin D (VA). This consists of 4 times weekly administered VCR followed by a 2 week rest period. actinomycin D is administered on weeks 1 and 4. These courses are given 4 times total. Total duration of chemotherapy is 22 weeks.
- Standard risk (subgroup B) (SR-B) patients will be treated with 4 cycles of ifosfamide, VCR and actinomycin D (IVA). One course consists of 1 ifosfamide administration in week 1, weekly VCR administrations during 3 weeks during first 2 courses and 3 weekly administrations during second two courses and 1 actinomycin D administration in week 1. This is followed by 5 courses of VCR and actinomycin (VA). Both drugs are administered in 3 weekly intervals. The total duration of chemotherapy is 25 weeks. These patients are in complete remission after initial surgery and therefore will not receive further local treatment.
- Standard risk (subgroup C) (SR-C) has the same chemotherapeutic regimen as SR-B in the first 4 courses. Then a second surgery is planned in week 13. After this, 5 more VA or IVA courses are planned. Also, in some cases radiotherapy is administered starting from the fifth course.
- In standard risk (subgroup D) (SR-D) patients, treatment is almost identical to SR-C patients, only all 9 courses consist of IVA courses and standard radiotherapy is administered starting from the fifth course.
- High risk (subgroup E, F and G) (HR-EFG) treatment is identical to SR-D treatment. When treatment is finished an assessment of local control is performed. Afterwards randomization will follow between stop of treatment or maintenance therapy with 6 courses vinorelbine/cyclophosphamide. One course consists of 28 days with administration of vinorelbine on day 1, 8 and 15 and daily cyclophosphamide.
- Very high risk (subgroup H) (VHR-H) treatment is identical so SR-D treatment, only doxorubicin is administered on days 1 and 2 of courses 1 through 4. Furthermore, after therapy an assessment of local control is done. This is followed by a maintenance therapy, which is standard care (no randomization) and identical to HR-EFG treatment.

In conclusion is VCR administered 16 times. All administrations have a 1,5 mg/m^2 dosage.

Use of co-intervention

Besides VCR, children with cancer are given other chemotherapeutic agents and radiotherapy during their treatment period as well. The most important ones are listed in paragraph 5.1.1. through 5.1.7.

Almost all patients are given concomitant medication that is not part of the anti-cancer therapy, but rather concerns infection prophylaxis, such as antibiotics and azole treatment (itraconazole, voriconazole, posaconazole en fluconazole). Furthermore, other supportive care is given, such as painkillers, anti-emetics, diuretics, granulocyte colony stimulating factor (GC-SF), anti-hypertension medication and suppletion of electrolytes. These medications might influence VCR metabolism. In any case, all medications known to have an effect on VCR metabolism will be carefully registered and will be taken into account when analyzing the data. Moreover, it is a standard clinical policy to refrain from VCR metabolism influencing medication on the day that VCR is given.

In clinical practice the VCR dosages are sometimes temporarily lowered or even discontinued because of severe PNP. Obviously, all protocol deviations concerning the VCR administrations will be carefully registered and taken into account when analyzing the data.

Escape medication

To date, no specific preventive or curative treatment is available to manage VCR-induced PNP. In some cases gabapentin or a similar drug is given in order to diminish neuropathic pain, but most often the only way to decrease PNP is to reduce VCR dosages, increase intervals, or discontinue VCR treatment, thereby impairing treatment efficacy. The use of medication such as gabapentin will be carefully registered.

INVESTIGATIONAL PRODUCT

Name and description of investigational product(s)

VCR is a vinca alkaloid obtained from the periwinkle plant Vinca rosea Linn. For more than 4 decades, it has been a key drug in the treatment of various childhood malignancies, including pediatric ALL, NB, SRMB, HRMB, LGG, HL and RMS. VCR is a mitotic inhibitor as it acts by inhibiting the assembly of microtubules into mitotic spindles. Hence, it is a cell-cycle-phase-specific cytotoxic agent which can exert its effect in a number of ways:

- by binding to a specific site of tubulin and by forming a tubulin-alkaloid aggregation complex;
- by binding to a high affinity site of tubulin, incorporated into microtubeles, and by inhibition of further incorporation of tubulin into the existing microtubule;
- by binding to a low affinity site on the microtubule wall that cases protofilament separation.

Summary of findings from non-clinical studies

The Dutch SPC as well as the “Public Assessment Report of the Medicines Evaluation Board in the Netherlands” concerning VCR are readily available and have been inserted in the Investigator Site File.

Summary of findings from clinical studies

The Dutch SPC as well as the “Public Assessment Report of the Medicines Evaluation Board in the Netherlands” concerning VCR are readily available and have been inserted in the Investigator Site File.

Summary of known and potential risks and benefits

The Dutch SPC as well as the “Public Assessment Report of the Medicines Evaluation Board in the Netherlands” concerning VCR are readily available and have been inserted in the Investigator Site File.

Description and justification of route of administration and dosage

In case a bolus injection is given the VCR is administered directly into a vein which, in most cases, takes 2 to 3 minutes. In order for a VCR injection to be classified as a bolus injection, the injection should take at least 1 minute and 5 minutes at maximum. In case a one-hour infusion is given the VCR is continuously infused for 60 minutes through a central venous catheter (see also chapter 11.4). All patients routinely get such a catheter. In the various protocols for treatment of pediatric ALL, NB, SRMB, HRMB, LGG, HL and RMS the dose of the VCR administrations is 1.5-2.0 mg/m^2 per administration, with a maximum of 2.0 mg per administration. These doses will also be applied for the participants in the current study.

Dosages, dosage modifications and method of administration

In this study the VCR treatment for all participants will be given according to the various treatment protocols. Thus, no changes will be made regarding the number, timing of doses of the VCR administrations, unless the medical situation of the participants requires to do so (see chapter 5.2 and 5.3).

Two administration methods of VCR are being compared: short-term infusions which take up to 1 minute to a maximum of 5 minutes per injection versus one-hour infusions which are given over 60 minutes per infusion.

Preparation and labelling of Investigational Medicinal Product

The medication for the current study is already being used for patient care purposes. The pharmacy will deliver either ready-to-use short-term infusions or ready-to-use infusion solutions for the study. The charge number of the flacon will be recorded by the pharmacy on the preparation prescription. The protocol of the VUmc pharmacy for preparation of the bolus injection as well as the one hour infusion of VCR has been attached (see attachments 15.1 and 15.2). In addition, an example of a label (in Dutch) has been attached (see attachment 15.3).

NON-INVESTIGATIONAL PRODUCT

Each of the study participants, regardless of randomization group, is treated according to the standardized protocols for the treatment of pediatric ALL, NB, SRMB, HRMB, LGG, HL and RMS. All these protocols are research-based protocols which encompasses a national or international multicenter (randomized) clinical trials with various study aims

Details of these different treatment protocols can be found at:

- ALL: ALL-11: https://www.skion.nl/voor-professionals/behandelrichtlijnen/protocollen/100/all-11/
  EsPhALL: https://www.skion.nl/voor-professionals/behandelrichtlijnen/protocollen/120/esphall/
- NB: https://www.skion.nl/voor-professionals/behandelrichtlijnen/protocollen/484/siop-wilms-2001/
- SRMB: https://www.skion.nl/voor-professionals/behandelrichtlijnen/protocollen/349/acns0331/
- HRMB: https://www.skion.nl/voor-professionals/behandelrichtlijnen/protocollen/415/acns0332/
- LGG: https://www.skion.nl/voor-professionals/behandelrichtlijnen/protocollen/340/siop-lgg-2004/
- HL: https://www.skion.nl/voor-professionals/behandelrichtlijnen/protocollen/134/euronet-phl-c1/
- RMS: https://www.skion.nl/voor-professionals/behandelrichtlijnen/protocollen/381/epssg-rms-2005/

METHODS

Study outcomes

Main study outcome

PNP will be evaluated by using the NCI Common Terminology Criteria for Adverse Events (CTCAE), version 4.03 (43). This is a widely-used scoring method for reporting adverse events in clinical trials. A grading (severity) scale is provided for each event. In this study, PNP will be evaluated by scoring peripheral motor neuropathy, peripheral sensory neuropathy, neuralgia (pain), and constipation. For motor and sensory neuropathy the scale ranges from 0 (no symptoms) to 5 (death); for neuralgia and constipation the scale ranges from 0 (no symptoms) to 3 (severely limiting ADL activities). In addition, a recently developed neuropathy measurement tool (ped-mTNS) (44) will be used to assess PNP. The ped-mTNS is a reliable and valid measure of chemotherapy-induced peripheral neuropathy in children with cancer and is associated with relevant functional limitations. It evaluates sensory symptoms, motor function, autonomic functions, light touch sensation, pin sensibility, vibration sensation, distal strength, and deep tendon reflexes. The ped-mTNS can be completed in 15 minutes.

Secondary study outcomes

Quality of life (QoL)

Health-related QoL will be measured by means of the Health Utilities Index Mark 3 (HUI3) and the Pediatric Quality of Life Inventory (PedsQL). The 15-question format of the HUI3 will be used (45). It consists of eight attributes (vision, hearing, speech, ambulation, dexterity, cognition, pain, and emotion), which can be described in 5 or 6 levels and describe the patient's health state. Attribute levels will be used to determine single-attribute utility (SAU) scores and multi-attribute utility (MAU) scores using published utility functions. Scores of 0.00 represent being dead and 1.00 living in perfect health. Filling out the HUI3 takes 5 minutes on average. The PedsQL is a validated and easy to use questionnaire which has been successfully used in childhood cancer populations (31). For ages 2-4 years, parent proxy reports will be used; for ages 5-18 years both the child and the parent will be asked to fill out the questionnaire. Generic QoL is measured with the PedsQL4.0 Generic Core Scales, which have been used in children with cancer before and takes about 4 minutes to complete (7, 46). Disease-specific QoL will be measured with the PedsQL Cancer Module. This instrument comprises of eight subscales: pain, nausea, procedural anxiety, treatment anxiety, physical attractiveness, worry, communication, and cognitive function and takes about 8-10 minutes to complete. Scoring is similar to the generic core scales. Parent-proxy and child self-reports are available and age-appropriate items are included. Both the Generic Core Scales and the Cancer Module has satisfactory psychometric properties.
Furthermore, during the study period of the current study, another study by Van Litsenburg et al. (47) will take place concurrently for the ALL patients participating in the current study. Since in this study the same type of inclusion criteria apply for the participants, a large proportion of the ALL patients might very well participate in both studies. Given the fact that both studies use the same quality of life measurement tools, the timing of the QoL measurements of the current study is scheduled in such a way that collected QoL data can be exchanged. By doing so, patients and/or parents will not be asked to fill out the same questionnaires in a short period of time.

Costs

The questionnaire to be used for the evaluation of costs is an adaptation of a well-tested questionnaire used by the institute for Medical Technology Assessment at the Erasmus Medical Centre Rotterdam (prof. dr. Carin Uyl) which has been used for cost-effectiveness analyses of cancer treatments in the past. For the current study a societal perspective will be taken into account when analyzing the costs involved in VCR-induced PNP following both

administration methods. This perspective implies that direct medical costs, direct non-medical costs, and indirect costs will be evaluated. Direct medical costs are the costs associated with the treatment of PNP, such as physiotherapist visits, medication to decrease neuropathic symptoms, and medical devices (wheelchair, braces, splints). The non-medical costs are those costs associated with the PNP treatment that have to be paid by the parents of the cancer patients, such as transport and parking costs, and costs for care of siblings. The indirect costs are costs due to loss of productivity of the parents. The direct medical costs will be calculated using data retrieved from medical records. The non-medical costs and costs outside the hospital will be evaluated by the use of a short questionnaire to be filled out by the parents (at three points in time). Unit costs will be collected from official national reimbursement systems. Costs of medications will be derived from the Z-index. The costs of a hospital day, outpatient visits, day care treatment, physiotherapist, general tests (such a laboratory testing, etc.) will be calculated by means of a cost price study. The base year for unit costs will be 2014. Costs will be expressed in Euros. As the time horizon is 6 months, no discount rate will be used. Since it is very unlikely that applying one-hour continuous infusion will lead to a worse clinical outcome when compared to a bolus injection, the economic outcome will only be expressed in terms of incremental costs between the two administration methods.

Treatment efficacy

Obviously, a change in administration method of VCR should by no means detriment the effectiveness of the anti-cancer treatment. Therefore, treatment efficacy will be thoroughly monitored during the study by evaluating residual disease (MRD) when possible, cumulative incidence of relapses, event-free survival (EFS) and overall survival (OS). These data concern readily available data which will be provided by the DCOG.

It should be noted however, that it is very unlikely that for the current study applying an one-hour continuous infusion will lead to a worse therapeutic index when compared to a bolus injection (see also chapter 8.7). In fact, a higher treatment efficacy is more likely for the following reasons:

- It has been suggested that prolonged exposure of tumor cells to VCR has a better therapeutic effect. For example, a relation has been demonstrated between tumor response and the pharmacokinetic profile of VCR in human tumor xenograft models (48).
- A long-term follow-up study showed that the clinical outcome of children with standard risk B cell precursor ALL is related to VCR pharmacokinetics as measured on day 1, with the anti-leukemic effect of VCR being positively correlated with the systemic exposure of VCR (49).
- Continuous administration of VCR after a conventional bolus dose (plus cyclophosphamide) does not result in significant neurotoxicity and seems to be a safe strategy to increase the systematic exposure (14).

Pharmacokinetics

Pharmacokinetics of VCR will be evaluated in both intervention groups by drawing seven or eight blood samples after the VCR has been administered at several occasions. This depends on whether the patient is hospitalized and the sample at 24 hours indeed can be taken. If a patient is not hospitalized, this sample at 24 hours will not be taken. In a previous study reported by Guilhaumou et al. population pharmacokinetics of VCR in pediatric cancer patients have been described (25). Based on these data a graph of concentration versus time has been simulated (figure 1).

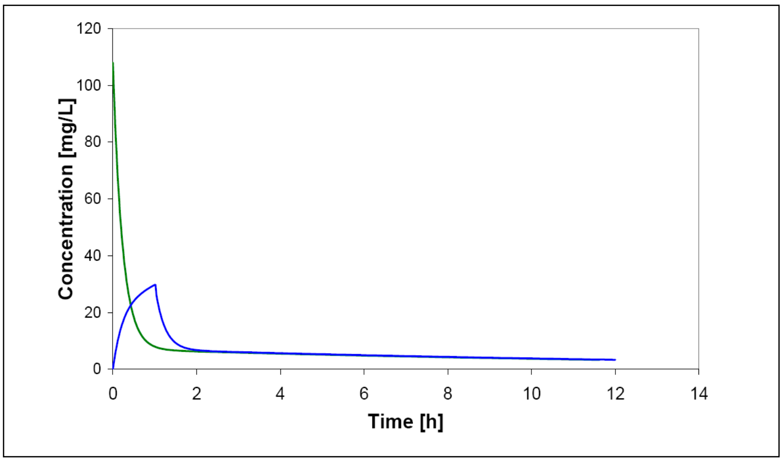

Figure 1: A simulation of VCR concentration versus time based on previous literature (25).

Based on these data the most informative sampling time points were calculated using the ADAPT II software (50). These are: 10, 20, 30, 40, 60, 75, 140 and 1440 minutes. Each sample will consist of 2 ml blood.
VCR plasma concentrations and the primary metabolite of VCR (M1) will be quantified in the plasma samples using a liquid chromatography-tandem mass spectrometry assay. All PK analyses will be performed by the Laboratory of Clinical Pharmacology and Pharmacy, VUmc.

Genotyping
To get further insight in the biological mechanisms underlying PNP we will collect DNA/RNA of the patients from normal blood cells at complete remission. White blood cells will be harvested from remission blood samples and pellets will be stored at -80°C. After all samples are collected, RNA and DNA will be isolated. We will screen polymorphisms that are currently associated with PNP and VCR sensitivity as shown in table 1 including any novel molecular variations that will become available during the project. We will determine the potential differences in these genetic variations between the patients with and without PNP and evaluate possible differences between the two intervention groups. These genetic analyses will be performed at the Hematology Laboratories of the Cancer Center Amsterdam in collaboration with the Department of Clinical Genetics.

Table 1A: Studies investigating associations between polymorphisms and vincristine-induced peripheral neuropathy

Author	CT	Treatment protocol	Target gene studied	Association with CIPN	Type of association	Number of patients
**Hartman (28)**	VCR	ALL-9	SNP's in CYP3A5, MDR-1 and MAPT	No		34 ALL survivors
**Renbarger (27)**	VCR	Unknown	CYP3A5/4	Yes	Unknown	113 ALL patients
**Egbelakin (21)**	VCR	AALL0331, AALL0232, AALL0434 AALL01P1	CYP3A5*3,*6,*7	Yes	CYP3A5 less CPIN then non CYP3A5	107 ALL patients >1 year treatment
**Johnson (31)**	THAL, VCR	Myeloma-IX, HOVON-50	Hypothesis driven SNP's	Yes	Rs2082382 and rs1042714 in ADRB2, rs7214723 in CAMKK, rs1934951 in CYP2C9, rs228832 in NFATC2, rs1555026 in ID3 and rs2301157 in SLC10A2	1495 patients with myeloma derived from 2 RCT's
**Plasschaert (51)**	VCR	VCR	MDR1 gene	No		70 newly diagnosed ALL patients
**Broyl (30)**	VCR, PS-341	HOVON-65/GMMG-HD4	Hypothesis driven SNP's	Yes	4 SNP's in PPARD, 2 SNP's in LTA, 1 SNP in the following: ABCC4, ABCC5, SLC10A2, ALDH1A1 and GLI1I (see reference for complete list)	9 grade 2-4 early onset VCR CIPN, 10 grade 2-3 late-onset VCR CIPN
**Moore (24)**	VCR	Multiple protocols	CYP3A5	No		50 multiple forms of cancer
**Diouf (52)**	VCR	St. Jude Total XIIIB protocol and COG AALL 0433	Genome wide association study	Yes	rs924607 in the promoter region of the CEP72 gene in	222 in St. Jude Total XIIIB protocol and 99 in

					chromosome 5	COG AALL0433

Table 1B: Studies investigating associations between polymorphisms and vincristine resistance

Author	CT	Treatment protocol	Association with CIPN	Type of association	Number of patients
**Schotte (53), Moqadam (54)**	PRDL, VCR, L-ASP, DNR	Not stated	Yes	miR125b, miR-99a and miR-100 to VCR and Dau, none to Pred, miR-454 to L-asp	81 newly diagnosed ALL patients, 17 control cases
**Dennison (22)**	VCR	Inapplicable	Yes	In CYP3A5 high-expressers CYP3A5 contributes 54-95% on VCR metabolism	56 HLM's
**Caronia (55)**	MTX, CDDP, ADR, VCR, CP	Surgery +MTX and alternate CDDP/ADR, CP and VCR to 48 months	Yes (measured by SNP's related to survival)	Rs4148416 in ABCC3, rs4148737, rs1128503, rs10276036 in ABCC1	102 patients with osteosarcoma

CT: Chemotherapy, CIPN: Chemotherapy Induced Peripheral Neuropathy, VCR: Vincristine, ALL: Acute Lymphoblastic Leukemia, SNP: Single Nucleotide Polymorphism, THAL: Thalidomide, HOVON: Hemato-Oncologie voor Volwassenen Nederland, PS-341: Bortezomib, PRDL: Prednisolone, L-ASP: L-asparaginase, DNR: Daunorubicin, miR: microRNA, HLM: Human Liver Microsomes, MTX: Methotrexate, CDDP: Cisplatin, ADR: Adriamycin, CP: Cyclophosphamide.

Randomization and treatment allocation

Patients will be randomly assigned to receive all VCR administrations of the various treatment protocols either by short-term infusions (group A) or by one-hour infusions (group B). Randomization will be done digitally through TENALEA portal. This will be done using block randomization to maintain balance between both groups. In order to ensure equal distribution of several key patient characteristics over the two intervention groups, blocked randomization will be used with patients being stratified according to age (2-10 years and 11-18 years), country treated (the Netherlands or Belgium), and sex. Random permuted blocks will be used within the stratification groups. This ensures that both administration methods are balanced at the end of every strata block.

Study procedures

The study is set up as a prospective, open-label, randomized controlled trial. Patients will be randomized into two groups. Patients in group A (the bolus group) will receive all VCR administrations during their treatment period by short-term infusions. Patients in group B (the infusion group) will receive all VCR administrations by one-hour infusions. The bolus injection will take up 1-5 minutes, whereas the one-hour infusion will be given over 60 minutes.

The different study measurements will be performed at several points in time: at baseline, one to five times during the two years of VCR-therapy (dependent of the length of VCR therapy) and 6 months after the end of VCR-therapy. Measurements of the various study outcomes will be scheduled in the following way:

Table 2: Overview of the study measurements

	**Timing**	**Outcome measurements**
**T0**	Before 1st VCR administration)	• PNP1
**T1**	After 4 VCR administrations:	• PK1<br>• QoL1<br>• PNP 2
**T 1.1**	During complete remisson/during treatment evaluation	• DNA collection
**T2**	At start reintroduction of VCR	• PNP2<br>• PK2<br>• QoL2<br>• Costs1
**T3**	When applicable during further VCR treatment (+/- after 10 VCR administrations since last measurement)	• PNP3<br>• QoL3
**T4**	When applicable during further VCR treatment (+/- after 10 VCR administrations since last measurement)	• None, only VCR administered
**T5**	When applicable during further VCR treatment (+/- after 10 VCR administrations since last measurement)	• PNP4<br>• QoL4<br>• Cost2<br>• TE
**T6**	6 months after end of VCR therapy (number of measurement depend on length of VCR treatment)	• PNP5<br>• QoL5<br>• Costs3<br>• TE

PNP = peripheral neuropathy; PK= pharmacokinetic measures; QoL= quality of life; TE = therapeutic effectiveness

In appendices 15.4 – 15.10 detailed schedules are given of the VCR administrations as well as the study measurements per included diagnosis.

The pharmacokinetic samples will be collected in the following way:
In order for the pharmacokinetic blood sampling to run smoothly all participants will be inserted an extra venous cannula which is to be inserted at time points on which the participants will undergo general anesthesia as prescribed by their treatment protocol. This allows the insertion of an extra intravenous cannula (which is needed for the PK measurements) with as little as possible extra burden for the patients.
The reasons for the anesthesia within the various treatment protocols include:

- a bone marrow or lumbar puncture with or without intrathecal medication (ALL patients).

- imaging techniques used for treatment evaluation used in NB, SRMB, HRMB, LGG, HL and RMS protocols in case the patient is younger than approximately 6 years.

When insertion of the extra venous cannula under general anesthesia does not take place according to the protocol, this part of the study becomes optional, giving parents and patients the opportunity to participate in the trial when the burden of inserting an extra venous cannula is considered too high. When the burden is acceptable to patients and parents they can choose to participate in the PK part of the study as well.
In ALL, where the time points of PK sampling are completely in line with bone marrow and lumbar puncture under general anesthesia, the PK part of the study remains integrated in the study.

Figure 3: PK measurements on a 'PK measurement day' outlined for both randomization groups.

1 hour infusion group		push injection group	
start VCR administration (60 min.)		start VCR adm. (5 min. max)	
	t=10 min: 1st sample		t=10 min: 1st sample
	t=20 min: 2nd sample		t=20 min: 2nd sample
	t=30 min 3rd sample		t=30 min 3rd sample
	t=40 min: 4th sample		t=40 min: 4th sample
	t=60 min: 5th sample		t=60 min: 5th sample
	t=75 min: 6th sample		t=75 min: 6th sample
	t=140 min: 7th sample		t=140 min: 7th sample
	t=1440 min: 8th sample; if possible*		t=1440 min: 8th sample; if possible*
** t=24 hours: the feasibility of this measurement will be evaluated case-by-case, as it depends on logistical/practical considerations. After n=10 evaluable samples of t=24 hours, it will be evaluated whether sampling at t=24 hours indeed is imperative and should be done for the remaining part of the participants.*			

As mentioned in figure 3, the PK sampling at t=24 (t=1440 minutes) hours will only be performed in case it is logistical/practical feasible. This means that the patient and parents are asked whether they are able to return to the hospital at t=24 hours or if they are already in the hospital. If it appears that this is not the case, the last PK sample will be considered to be missing. Moreover, after 10 evaluable PK samples from 10 different patients at 24 hours, it will be evaluated whether sampling at t=24 hours indeed is imperative for the estimation of the clearance of VCR and should be done for the remaining part of the participants.
The blood sample for DNA genotyping will be drawn approximately halfway through the total treatment period. Treatment efficacy will be evaluated using data that are already collected by the DCOG according to the treatment protocols.

Withdrawal of individual subjects
Subjects can leave the study at any time for any reason if they wish to do so without any consequences. The investigator can decide to withdraw a subject from the study for urgent medical reasons.

Replacement of individual subjects after withdrawal

Enrolled subjects are all subjects who signed the informed consent form. Evaluable subjects are all subjects who received at least 4 administrations of VCR (either through bolus injection or 1 hour infusion) followed by a PNP assessment. Data analyses will be performed on the dataset of all evaluable subjects. Subjects will be enrolled until a total of 88 evaluable subjects is reached.

Follow-up of subjects withdrawn from treatment

Treated subjects are all subjects who received at least at least 4 administrations of VCR as part of the study protocol. Demographic and baseline characteristics and safety analyses will be performed on all treated subjects.

Premature termination of the study

It is very unlikely that for the current study applying an one-hour continuous infusion will lead to worse treatment efficacy when compared to a bolus injection. It is more likely to be the opposite. Continuous infusion of chemotherapeutic drugs may result in better therapeutic outcomes compared to short-term infusions, especially if the drug half-life is short and the drug activity is cell cycle dependent, which is the case for VCR. Furthermore, prolonged exposure of tumor cells to VCR seems to have a greater impact on tumor response as evaluated in human xenograft models (48). Moreover, it has been demonstrated that continuous infusion of VCR *after* a conventional bolus dose (plus cyclophosphamide) did not result in significant neurotoxicity and appeared to be a safe strategy to increase systemic exposure (14). In order to warrant optimal therapeutic effectiveness for all study participants, an interim analysis will be performed once a year, examining whether the cumulative relapse rate, EFS, or the relapse incidence is not adversely affected for the one-hour infusion group. If it seems that this is the case, the study will be prematurely terminated. On the other hand, it seems unlikely that in view of the excellent overall outcome and the limited number of patients, therapeutic outcome will be significantly better in the one-hour infusion group.

The principal investigator can decide to prematurely terminate the study on the following criteria:

- There is evidence of an unacceptable risk for trial subjects (i.e. safety issue)
- There is reason to conclude that it will not be possible to collect the data necessary to reach the study objectives and it is therefore not ethical to continue enrolment of more patients. The PI will notify the METC and the competent authority within 15 days, including the reasons for the premature termination.
